# Spatial FBA reveals heterogeneous Warburg niches in renal tumors and lactate consumption in colorectal cancer

**DOI:** 10.1038/s41540-026-00654-x

**Published:** 2026-01-27

**Authors:** Davide Maspero, Giovanni Marteletto, Francesco Lapi, Bruno G. Galuzzi, Irene Ruano, Ben Vandenbosch, Ke Yin, Sabine Tejpar, Alex Graudenzi, Holger Heyn, Anna Pascual-Reguant, Chiara Damiani

**Affiliations:** 1 Centro Nacional de Análisis Genómico, Barcelona, Spain; 2 Immunobiology Department, Yale School of Medicine, New Haven, CT USA; 3 Department of Biotechnologies and Biosciences, University of Milano-Bicocca, Milan, Italy; 4 Institute of Bioimaging and Complex Biological Systems (IBSBC), Segrate, Italy; 5 SYSBIO Centre of Systems Biology/ISBE.IT, Milan, Italy; 6 National Biodiversity Future Center (NBFC), Palermo, Italy; 7 Department of Oncology, KU Leuven, Leuven, Belgium; 8 Department of Informatics, Systems and Communication, University of Milano-Bicocca, Milan, Italy; 9 Universitat de Barcelona (UB), Barcelona, Spain

**Keywords:** Cancer, Computational biology and bioinformatics, Oncology

## Abstract

To investigate how spatial constraints shape cancer metabolism, we devised the spatial Flux Balance Analysis (spFBA) framework for the enrichment of spatial transcriptomics data with relative estimates of metabolic fluxes. Applying spFBA to newly generated high-resolution datasets of paired primary colorectal tumors (CRC) and liver metastases revealed lactate consumption in both primary and metastatic regions. The presence of lactate-consuming niches was confirmed in an independent public dataset, suggesting this may be a recurrent metabolic feature of CRC. Importantly, application to public datasets of renal cancer showed widespread lactate production, consistent with a dominant but heterogeneous Warburg phenotype, ruling out general prediction biases or algorithmic artifacts. spFBA also consistently identified regions of increased proliferation across datasets, supporting the biological validity of its predictions. The framework is applicable to any sequencing-based spatial dataset to effectively uncover metabolic programs that remain invisible to gene expression analysis alone.

## Introduction

Spatial organisation is a fundamental principle of tissue physiology and pathology. In cancer, gradients in oxygen, nutrients, and signaling factors create heterogeneous microenvironments that shape cell behaviour and metabolic function. This spatial heterogeneity is particularly relevant in tumors, where metabolic adaptations support proliferation^1–4^, immune evasion, and metastasis. Yet, our understanding of how metabolism is organized across tissue architecture remains limited, largely because existing tools for metabolic analysis lack spatial resolution.

While techniques like mass spectrometry imaging and multiplexed immunohistochemistry have begun to map metabolic markers in situ^5–7^, they are limited in scope and throughput. In contrast, spatial transcriptomics (ST) enables genome-wide measurements of gene expression across intact tissue sections, providing a rich resource to reconstruct cellular functions, including metabolism, in their native spatial context.

This gap underscores the need to derive metabolic fluxes from gene expression data computationally. Although factors beyond transcriptional regulation influence metabolic fluxes, successful inference approaches have been developed using bulk^8^, single-cell^9,10^, or clustered transcriptomic data^11^. The constraint-based (CB) modeling framework is the most established approach for simulating feasible metabolic flux distributions under physico-chemical constraints^12^. Although self-supervised learning approaches that relax mass balance constraints are emerging as promising alternatives^13^, they currently do not account for reactions not directly linked to gene expression, such as oxygen uptake or the biomass synthesis pseudo-reaction, both critical readouts in metabolic analysis. To our knowledge, no approach has yet been applied to model fluxes at the level of spatial spots.

Currently, the lack of spatially resolved flux measurements prevents the quantitative validation of spatial fluxomics predictions against ground-truth data. This limitation is common to all approaches in the field of metabolic flux inference, where benchmarking efforts are typically restricted to specific, well-characterized pathways, (i.e., glycolysis) rather than evaluating global flux distributions^9,13^. However, ST opens new opportunities: histological structure provides a natural reference frame to assess whether biologically meaningful spatial patterns emerge in the predicted flux distributions.

To address these challenges, we focused on a clinically relevant context: the metabolic rewiring of colorectal cancer (CRC) during liver metastasis. CRC is among the most common malignancies worldwide, and its metastatic progression is strongly linked to metabolic plasticity. We sought to determine whether primary tumors and matched metastases exhibit distinct spatial metabolic patterns, and whether ST can uncover meaningful differences at the flux level.

To this end, we generated spatial transcriptomic datasets from a single patient–derived primary colorectal tumor and two paired liver metastases. The datasets were generated with stereo-seq to have the high-resolution required to investigate tumor-stroma interactions in vivo. To analyze these data, we extended our previous work on integrating bulk^14^ and single-cell RNA-seq data into CB metabolic models^10,15^ to the spatial domain. The resulting approach, which we term spatial Flux Balance Analysis (spFBA), enables the simulation of metabolic activity while accounting for transcriptionally encoded substrate availability. spFBA enhances the expressive power of gene set enrichment frameworks by capturing reaction directionality, predicted growth rates, and preferences for nutrient uptake or secretion. As a result, it generates a robust, reaction-level enrichment map for each spatial spot.

## Results

### The spatial FBA approach

To extract spatially resolved metabolic activity from transcriptomic data, we developed the Flux Balance Analysis (spFBA) framework, rooted in CB metabolic simulation^16^.

spFBA takes as input a metabolic network reconstruction and ST data, and returns a matrix of Flux Enrichment Scores (FES) values for each reaction across spatial spots. These scores capture the direction and relative magnitude of reaction usage, enabling the reconstruction of local metabolic activity under steady-state constraints.

Unlike classical FBA approaches, like Parsimonious FBA, which rely on optimizing a predefined objective function (typically biomass production)^17^, spFBA adopts a flux sampling approach. Flux sampling has become an established strategy in CB modeling to explore the range of feasible behaviors supported by a given metabolic network^18^. By extending this approach to the spatial domain, spFBA does not impose a common metabolic goal across spatial locations. Instead, it allows each spot to explore its own locally constrained solution space, guided by transcriptional profiles and encompassing both proliferative and quiescent metabolic states.

Operationally, spFBA combines our recently developed flux sampling strategy^19^, designed to minimize the risk of false discoveries due to undersampling, with spatially aware gene expression constraints. The integration strategy, similar to the one we previously employed in scFBA^10^, is based on differential gene expression across spatial spots. This approach avoids hard thresholds^20,21^ and enables meaningful comparisons of metabolic activity across regions, preserving subtle spatial differences without imposing unrealistic assumptions on absolute flux magnitudes, as in expression-based flux optimization methods^9,22^.

Although we previously modeled metabolic interactions between single cells in well-controlled systems, where extracellular fluxes and medium composition were known^10^, we deliberately chose not to model explicit spatial exchanges between spots in this study. In clinical datasets, no direct information is available on nutrient availability, exchange surfaces, or the identity of neighboring regions. Introducing such assumptions could override informative spatial signals encoded in the transcriptomic data. Our agnostic approach allows spatial organization to emerge from the data itself. Each spot is modeled independently, and we assess *a posteriori* whether coherent metabolic patterns arise, reflecting diffusion, microenvironmental gradients, or tissue-level architecture.

We previously observed that the emergence of flux patterns coherent with gene expression is not guaranteed when transcriptomic data are integrated into CB models^15^. On the contrary, when using raw expression matrices—without proper denoising or smoothing—the resulting flux distributions often appear noisy and spatially unstructured, despite strong organization in the underlying gene expression. This highlights a core property of spFBA: due to the reduced degrees of freedom imposed by steady-state constraints, it remains agnostic to transcript-flux relationships, which are instead explicitly encoded, e.g., in machine learning models like scFEA^13^.

Starting from a metabolic network model and assumptions about nutrient availability, spFBA first applies Flux Variability Analysis (FVA), without imposing any optimization objective, to define the space of feasible flux distributions under steady-state conditions. Properly preprocessed ST data are then used to compute a Reaction Activity Score (RAS) for each reaction in each spot. Differences in RAS across spots define local subregions of the feasible space.

These subregions are then sampled extensively. The resulting flux distributions are aggregated into normalized centroids, yielding a final FES for each reaction in each spatial spot.

**Fig. 1:**
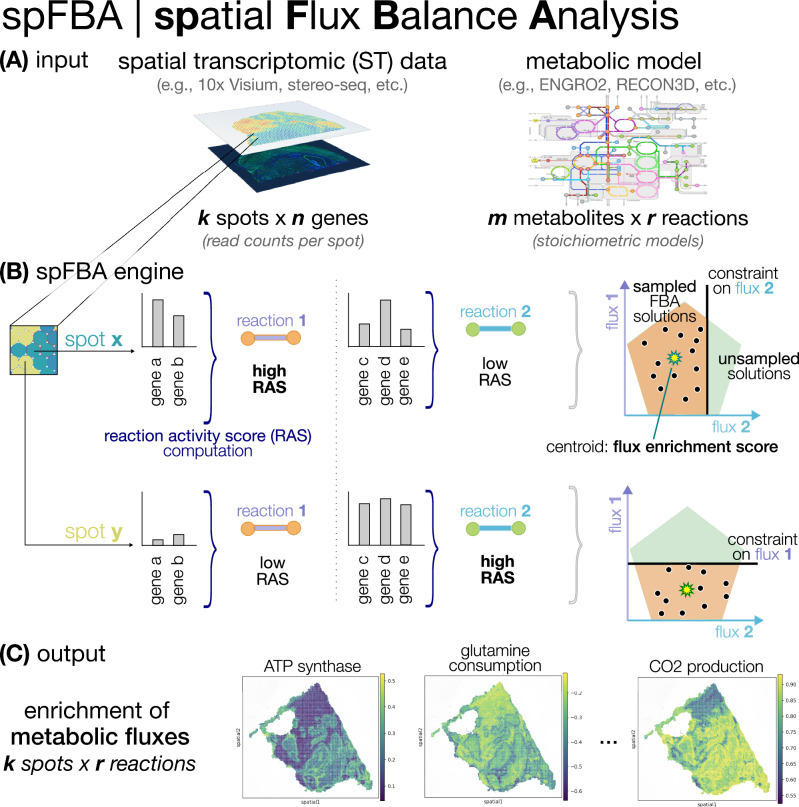
Overview of spFBA. **A** spFBA takes two data types as input: (1) a spatial transcriptomic (ST) read counts matrix of a biological sample, in the form of a *k* spots per *n* genes matrix; (2) a metabolic network reconstruction, either core (e.g., ENGRO2^14^) or genome-wide (e.g., RECON3D^23^), in the form of a *m* metabolites per *r* reaction matrix. **B** After preprocessing via standard pipelines (see “Methods”), our framework computes for each reaction in each spot a reaction activity score (RAS), based on the expression level of the genes involved in the reaction. The RAS is then employed to set reaction-specific constraints in the Flux Balance Analysis (FBA) computation. Specifically, a high number of FBA solutions are randomly sampled in the constrained region, for each reaction, the flux enrichment score is defined as the centroid of the distribution. **C** spFBA returns as output the flux enrichment scores, in the form of a *k* spots per *r* reactions matrix. Example heatmaps of three reactions are displayed, namely ATP synthase, glutamine consumption, and CO_2_ production.

The spFBA framework is schematized in Fig. 1.

From a computational standpoint, spFBA is compatible with genome-scale metabolic models (e.g., RECON3D^23^). In this study, we deliberately chose the manually curated core model ENGRO2^14^, as its streamlined structure improves controllability and interpretability.

### spFBA well recapitulates the tissue architecture

Since broad metabolic differences between tumors and healthy tissues are well established, we first assessed the validity of spFBA using a publicly available ST dataset of renal cancer with tumor-normal interface regions^24^. Clear cell renal cell carcinoma (ccRCC), which consistently displays a Warburg phenotype with elevated glycolysis and lactate production^25^, provides an ideal setting to test whether spFBA recovers expected tumor-normal metabolic contrasts. Importantly, using a tumor distinct from CRC also allows us to verify that spFBA produces context-specific, rather than generic or tissue-independent, predictions.

**Fig. 2:**
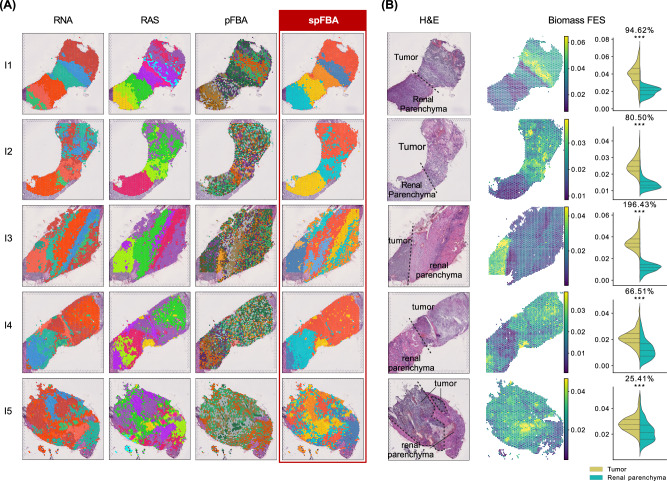
spFBA validation on ccRCC samples. **A** clustering results obtained with different data layers: RNA (preprocessed reads counts), RAS, pFBA, and spFBA. Color scales were independently assigned to clusters, so they are not comparable across layers or samples. Sample information is provided in Table 1. **B** From left to right: annotated H&E staining taken from^24^; Biomass FESs for each spot obtained with spFBA; Statistical comparison of the biomass FESs between spots annotated as tumor and parenchyma. The violin plot titles indicate the percentage of variation in the averages of the two populations and the results of *t*-tests. Statistical significance is reported using a star notation system, with a significance threshold of 0.05. When applicable, the same information for the ccRCC tumor core samples is in Supplementary Fig. S1.

As a minimal preliminary check, we examined whether regions with similar gene expression patterns also exhibit comparable flux distributions. While it is reasonable to expect that metabolically similar cells activate corresponding genes, as motivated above, this alignment is not guaranteed due to the constraints imposed by steady-state modeling. To this end, we compared clustering results obtained using three distinct feature sets, each representing a different biological layer: gene expression (RNA), RAS, and FES. Clustering parameters were independently optimized for each layer to ensure a fair comparison. As qualitatively evident (Fig. 2), the clusters derived from the FESs exhibit a coherent spatial organization that closely aligns with the histological architecture reflected in the transcriptomic data. Notably, this spatial coherence emerged despite the fact that no spatial coordinates were used in the modeling, which relied solely on transcriptomic content at the single-spot level. As expected, the number of clusters observed in the fluxomics layer was generally lower than in the transcriptomic layer, due to the reduced degrees of freedom imposed by steady-state constraints, as previously reported in ref. ^10^. Although Fig. 2A focuses on interface samples for narrative clarity, similar patterns were also observed in core tumor regions (Supplementary Fig. S1).

To assess the added value of using flux sampling instead of optimization-based methods, we recomputed the flux layer using the same transcriptionally informed flux bounds, but replaced the sampling step with parsimonious FBA (pFBA), which selects the flux distribution that maximizes biomass production. As shown in Fig. 2A, clustering based on pFBA-derived fluxes clusters derived from pFBA fluxes appeared more scattered and less consistent with the underlying histological structure, compared to those obtained via sampling.

To quantitatively assess this advantage across all ccRCC samples (including core tumors), we compared the clustering outcomes derived from spFBA and pFBA layers against those obtained from RNA expression, using the mean V-measure across all ccRCC samples as a similarity metric. The V-measure, an entropy-based score for evaluating clustering quality with respect to a reference classification^26^, was substantially higher for the spFBA layer (*V* = 0.58) than for the pFBA layer (*V* = 0.28). A Mann-Whitney U test confirmed that the mean V-measure was significantly higher for spFBA compared to pFBA (*p* < 0.05).

Overall, these results further support our claim that alignment between transcriptional information and transcriptomics-informed flux predictions cannot be taken for granted. Notably, although both spFBA and pFBA used the same preprocessing and integration pipeline, the optimization approach failed to preserve key spatial information that was retained through sampling.

### spFBA detects cancer enhanced metabolic growth

Having shown that spFBA captures the transcriptomically defined tissue architecture, we next focused on the added value of spFBA: the ability to probe reactions not directly linked to gene expression, such as oxygen uptake and biomass production. To verify that metabolic growth rates are enriched in the tumor region, we used the FES of the biomass pseudo-reaction as a proxy. We compared the biomass FES between tumor regions and renal parenchyma in interface samples from five patients profiled in ref. ^27^. While the authors had annotated tumor and healthy tissue regions for samples I1 and I2, where the tumor-normal boundary is most distinct, we extended these annotations to the remaining interface samples. Fine-grained pathological annotations to accurately exclude non-tumoral regions were not feasible. This limitation reflects the constraints of working with low-resolution datasets, where distinguishing fine structural details is challenging.

Figure 2 B presents the annotated H&E staining alongside the biomass FESs for the interface samples and the frequency distributions of spots annotated as tumor or normal. The data reveal a clear and statistically significant separation between the two regions, with higher biomass enrichment consistently observed in the tumor region.

### spFBA captures the Warburg effect in ccRCC

Using spFBA, we characterized spatially resolved metabolic reprogramming in ccRCC, focusing on the Warburg effect. This metabolic phenotype, described a century ago^28^, involves preferential energy generation through glycolysis followed by lactate fermentation, even in the presence of oxygen.

In addition to glucose, lactate, and oxygen, we examined the exchange flux of other key metabolites, including glutamine^29^, glutamate^29^, serine^30^, glycine^31^, and palmitate, which has been implicated in metastasis^32^.

**Fig. 3:**
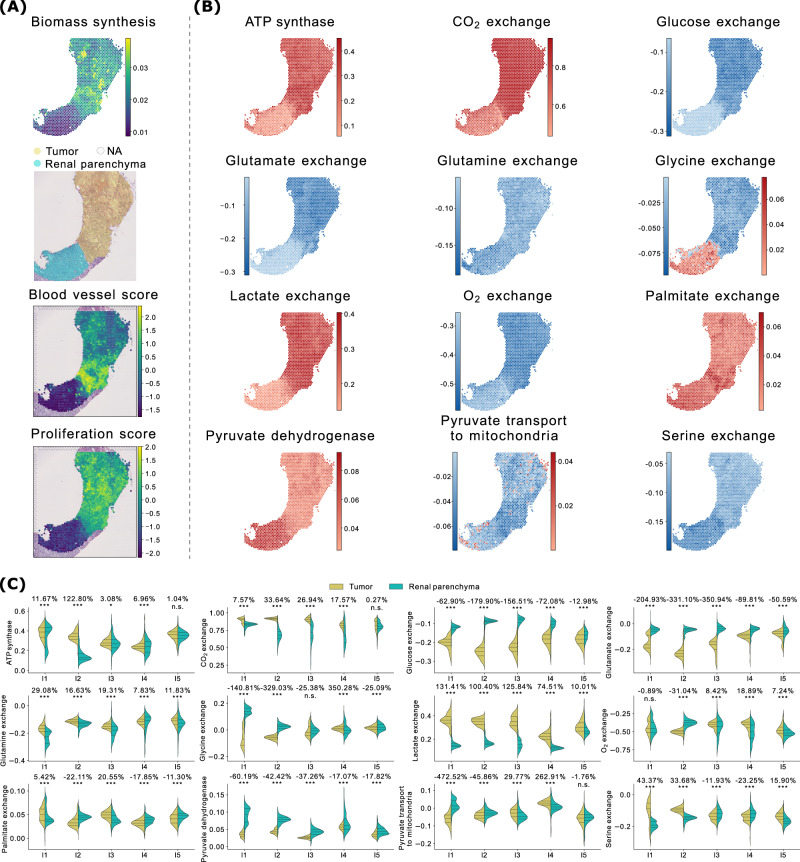
Tumor vs. parenchyma. **A** ccRCC sample I2: Biomass FES, annotated H&E image, and blood and proliferation scores derived from gene expression. The same information for the other samples is reported in Fig. 4 (patient PD47171), and in the supplementary material for all other kidney samples (Figs. S2, S3, S4, S5, S6, S7, and S8.) **B** FES of a set of reactions of interest (in alphabetical order) for ccRCC sample I2. For exchange reactions, negative values correspond to metabolite consumption, positive values to production. The same information for the other samples is available in Fig. 4 and in the supplementary figures mentioned above. **C** Statistical comparison of the FESs for the reactions in panel B between spots annotated as tumor and parenchyma, for all ccRCC interface samples. Violin plot titles indicate the percentage variation between the group averages and the results of *t*-tests. Statistical significance is reported using a star notation system (threshold: *p* < 0.05).

Figure 3 presents the spatial distribution of the FESs for the reactions under study in a representative interface sample (I2), along with a statistical comparison of these FESs between spots annotated as tumor and renal parenchyma, across all interface samples. We remark that, conventionally, intake fluxes are represented with a negative sign, meaning that a more positive distribution reflects lower consumption.

A striking observation is the markedly higher glucose consumption and lactate production in the tumor region (corresponding to the upper area, as per annotated H&E staining image in Fig. 3A), as expected under the Warburg effect. This difference in glucose consumption and lactate production rates was substantial and statistically significant consistently in all interface samples (Fig. 3B, C). Interestingly, oxygen consumption rates are generally similar between tumor and normal regions (Fig. 3C). In most samples, oxygen usage in tumor areas is only slightly reduced, with decreases remaining below 20%. However, the distributions are non-normal and highly variable, making these differences statistically uncertain. A notable exception is sample I2, where oxygen consumption increases by approximately 30% in the tumor region.

To investigate why tumor regions displayed sustained oxygen consumption despite also producing lactate, we analyzed blood vessel marker expression across spatial spots. The analysis revealed that tumor areas in interface sample I2 are indeed well vascularized, even more than the adjacent normal tissue (Fig. 3A), consistent with the elevated oxygen uptake predicted by spFBA.

This pattern of oxygen consumption, alongside enrichment in other metabolic indicators such as CO_2_ production and ATP synthesis (Fig. 3B, C), suggests that cancer cells adopt a hybrid respiratory-fermentative metabolism. This supports the idea that cancer cells are generally more metabolically active and ‘greedy’. One might be tempted to interpret lactate production in this scenario as a simple result of oxygen limitation, where excess glucose that cannot be oxidized is fermented into lactate. However, the situation portrayed by spFBA is far more complex. To explore the interplay between glycolysis and mitochondrial activity, we examined the FES for pyruvate dehydrogenase, which controls the entry of pyruvate into the TCA cycle. Remarkably, pyruvate dehydrogenase activity is significantly reduced in tumor regions across all interface samples, indicating that glucose-derived pyruvate is not oxidized via the TCA cycle. Instead, pyruvate transport to the mitochondria is negligible in the renal parenchyma and becomes negative in tumor regions, suggesting a reversal of transport from mitochondria to the cytosol. However, the frequency distribution of the FES for this reaction straddles zero, indicating that negative values may result from probabilistic variation rather than definitive biological reversal. In sample I2, the analysis of the confidence interval for the FESs reveals that the lower and upper bounds (Figs. S9 and S10) are nearly identical across most regions, indicating minimal uncertainty in the estimates. Nevertheless, a few scattered spots in the normal region show subtle shifts in the pyruvate transport FES from blue to red. This suggests that while reverse transport is generally negligible, localized variations may occasionally result in fluxes being classified as negative instead of positive.

Although this effect is limited and does not change the broader conclusion that cancer cells bypass canonical mitochondrial pathways, it underscores the need to carefully interpret fluxes near zero.

### Spatially distinct Warburg phenotypes reflect regional vascularization within the same tumor

The metabolic configuration revealed by spFBA at the tumor-normal interface aligns with the “selfish cell” interpretation of the Warburg effect^33^, in which cancer cells prioritize nutrient uptake to fuel their metabolic needs without suppressing mitochondrial respiration.

In a previous theoretical study^34^, it was shown, using a minimal metabolic model, that when oxygen availability is insufficient to fully oxidize available carbon sources, a strategy combining glutamine utilization via reductive carboxylation and conversion of most glucose into lactate provides a growth advantage. spFBA reproduced a similar pattern in our data, using the more detailed ENGRO2 network, which includes all essential and non-essential amino acids. Specifically, we observed high oxygen consumption alongside low pyruvate dehydrogenase activity, consistent with partial respiratory flux repression. Notably, across all interface samples, spFBA did not predict significant glutamine uptake in tumor regions; instead, it revealed consistent enrichment of glutamate consumption (Fig. 3).

**Fig. 4:**
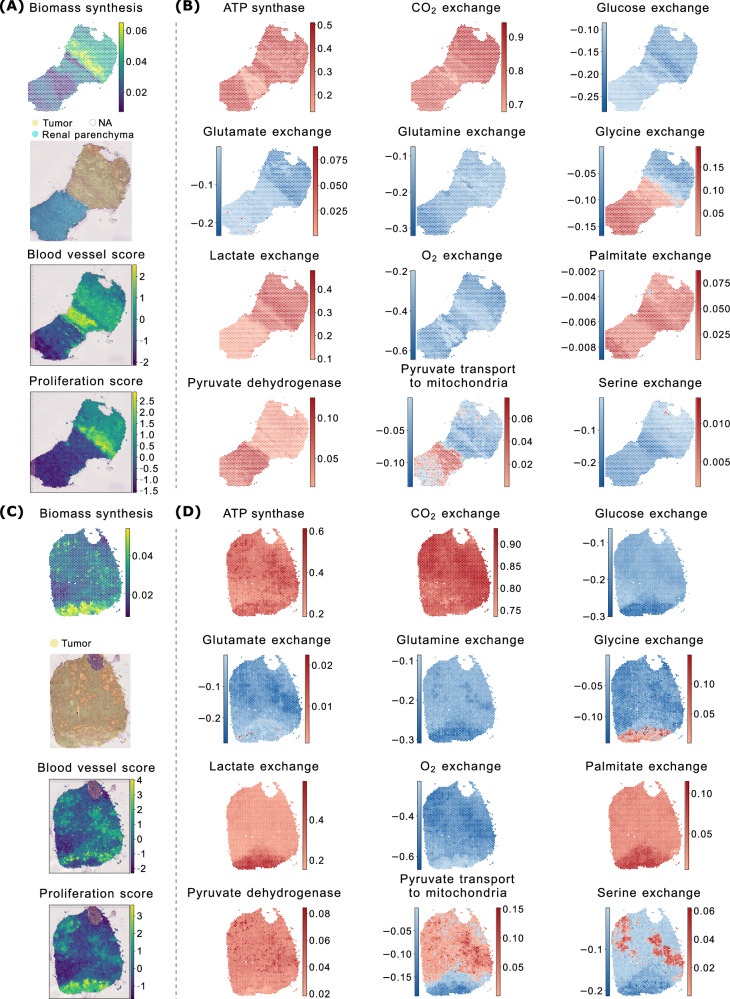
Paired interface and tumor core samples. **A** ccRCC interface sample I1: Biomass FES, annotated H&E image, and blood and proliferation scores derived from gene expression. **B** FESs for a set of reactions of interest in sample I1. In exchange reactions, negative values indicate consumption, positive values production. **C**, **D** Show the same analyses for the paired core sample C1.

The “selfish cell” scenario contrasts with the classical Crabtree effect^35^, in which oxidative phosphorylation is actively repressed. Interestingly, we identified metabolic signatures consistent with the Crabtree effect in tumor core regions. For instance, when we compared the interface sample I1 (Fig. 4A, B) to its paired core sample C1 (Fig. 4C, D), we noticed that the bottom part of the tumor core (Fig. 4D) shows increased lactate secretion and higher glucose uptake, but reduced oxygen consumption, indicative of a more fermentative phenotype. This region also exhibits enhanced biomass production, consistent with a Warburg-like growth strategy. We verified that, at the gene expression level, the proliferation score, computed by aggregating the expression of non-metabolic markers of proliferation (see Methods), is indeed substantially enriched in that area (Fig. 4A).

Unlike the tumor interface (Fig. 4B), where glutamate consumption is prominent, the fermentative region in the tumor core (Fig. 4D) lacks such enrichment. Instead, it tends to secrete glutamate while increasing glutamine uptake, suggesting a shift in nutrient utilization under differing microenvironmental conditions.

To investigate whether these differences in oxygen consumption reflect variations in local vascularization, we computed a vascularization score based on the expression of blood vessel gene markers. The spatial distribution of this score clearly indicates that the tumor region in interface sample I1 is more vascularized than the adjacent normal tissue (Fig. 4A), where spFBA predicts simultaneous high oxygen consumption and lactate production. Conversely, the bottom region of tumor core sample C1—which displays high lactate production and reduced oxygen consumption—corresponds to a poorly vascularized area (Fig. 4C).

This spatial overlap between hypoxic regions and areas with low vascularization further supports the biological relevance of spFBA predictions.

Collectively, these findings support the validity of spFBA in capturing spatially heterogeneous metabolic behaviors in ccRCC. Distinct Warburg phenotypes appear to coexist within the same tumor, shaped by local vascularization. At well-vascularized interfaces, the Warburg effect is accompanied by sustained oxidative phosphorylation, consistent with the hybrid metabolic phenotype described in some cancer contexts^36^. In contrast, tumor cores contain hypoxic zones with more classical fermentative profiles, in line with the Crabtree effect.

### scFBA can enrich metabolic interactions

A key advantage of scFBA over standard enrichment frameworks is its ability to identify spatial spots exhibiting opposing signs for exogenous nutrients, negative for consumption and positive for production. This feature is critical for uncovering metabolic cooperation within tissues.

For instance, in both interface sample I1 and its paired tumor core sample C1 (Fig. 4), as well as in interface I2, distinct regions can be observed where glycine is either produced or consumed. Specifically, one region tends to produce this metabolite, while another tends to consume it.

This phenomenon is particularly pronounced in the tumor core C1, where the glycine-producing region overlaps with an area of lower oxygen consumption. This overlap suggests a potential link between glycine production and metabolic adaptation to the tumor microenvironment, such as reduced oxygen availability.

While serine also shows regions with opposing metabolic signs in C1 (Fig. 4), the positive regions (highlighted in red) overlap with large congested blood vessels and multifocal areas of hemorrhage, as indicated by the annotated H&E staining in Fig. 4C (a cleaner version without annotations is also available as Supplementary Fig. S1). These histological features may affect the reliability of the results, potentially reflecting tissue-specific artifacts rather than genuine metabolic patterns.

It is important to note that we cannot confirm whether these populations exchange nutrients among themselves or only with the plasma. Determining this would require experimental measurements of plasma nutrient consumption and secretion rates, which are challenging to obtain in vivo. Additionally, a population mass balance constraint must be applied, as suggested in ref. ^10^. However, the absence of regions exhibiting lactate consumption enables us to rule out metabolic cooperation involving lactate in all ccRCC samples.

Notably, with only a few exceptions, most regions across all ccRCC samples do not consume exogenous lipids. Instead, they tend to secrete them, indicating a reliance on de novo lipogenesis—even in excess—to meet the metabolic demands of growth, as reported for many cancer cells^37^.

### CRC liver metastasis mimics tissue-of-origin metabolic traits

When applying spFBA to our CRC samples, we used the very same experimental settings as for ccRCC, ensuring that any differences observed in the results are solely due to variations in the ST information.

**Fig. 5:**
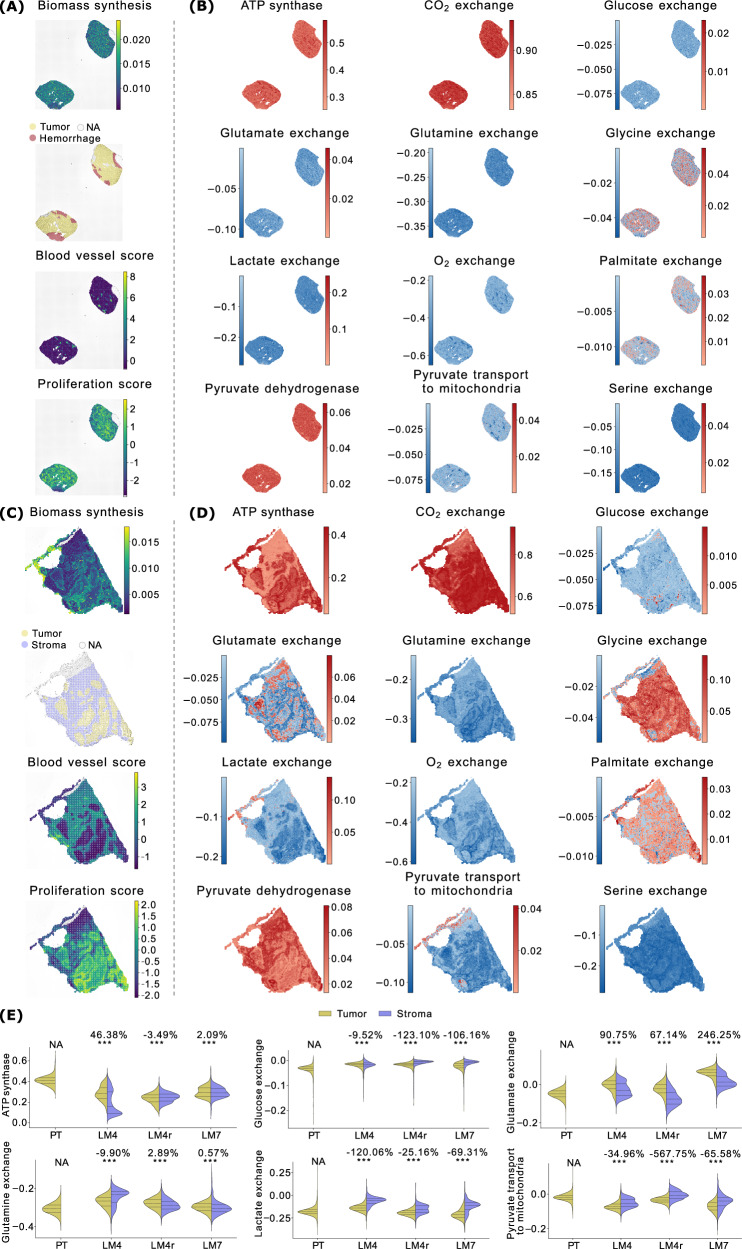
Primary tumor vs metastasis. **A** CRC PT sample: biomass FES, annotated H& E image, blood, and proliferation scores. **B** FESs for a set of reactions (in alphabetical order) in the PT sample. For exchange reactions, negative values indicate consumption, positive values indicate production. **C** As in panel A, for liver metastasis sample LM4. **D** As in panel B for sample LM4. **E** Violin plots show distributions of FESs in tumor and stroma spots; the titles report the percent difference in means, *p*-values from *t*-tests, and significance stars (threshold *p* < 0.05). Results for LM4r, LM7 are in Figs. S12, S13. The tumor vs stroma comparisons for all reactions in B are in Fig. S11.

It is particularly striking that, while lactate secretion was consistently detected by spFBA in all ccRCC samples, none of the CRC samples showed any lactate secretion (Fig. 5). On the contrary, most regions in all CRC samples exhibited an enriched lactate uptake.

We hypothesize that more exogenous lactate is available for both tumor and non-tumor cells to consume in the colon compared to the kidney. This may be due to the presence of various bacteria in the colon capable of producing lactate^38^. In contrast, in the kidney, exogenous lactate is likely less abundant, as supported by literature reporting that kidney cells tend to produce rather than consume lactate^39^.

Remarkably, lactate uptake was observed both in CRC primary tumor (PT) and in liver metastasis (LM) (Figs. 5 and S12, S13). The liver is one of the main organs in charge of lactate metabolism, which presents a net uptake of lactate^40^. Therefore, enhanced lactate consumption by metastatic tumor cells might be supported by the pre-existent context of lactate accumulation in physiological conditions. The common utilization of lactate by PT and LM highlighted by scFBA supports the recent hypothesis that differences in tissue nutrient availability might constrain the tissue-of-origin-shaped metabolism of cancer cells to limit sites of metastatic colonization^41^. Metastatic migration of colon cancer cells could depend on lactate accessibility of the destination tissue, which enhances the metabolic plasticity of tumor cells to react to changes in nutrient availability, thus maximizing cellular proliferation and growth.

### Differential stroma-tumor flux analysis highlights CRC tumor lactate consumption via non-canonical pathways

High-resolution ST allowed us to resolve differences in metabolic fluxes between tumor cells and the surrounding stroma in CRC liver metastases.

Focusing on lactate metabolism, the FES maps of lactate exchange in the representative metastatic samples (Fig. 5) highlight dark blue patches corresponding to tumor regions, surrounded by lighter areas representing stromal tissue. This spatial contrast suggests that tumor cells exhibit elevated lactate consumption, whereas stromal cells tend to release small amounts of lactate. The statistical comparison between annotated stromal and tumor regions (Fig. 5E) confirms that lactate uptake is significantly enriched in tumor areas across all CRC samples. Conversely, stromal regions show low levels of lactate uptake and occasional lactate secretion. Notably, lactate consumption appears more pronounced in liver metastases than in primary tumors, where the predominance of cancer cells and lack of clearly defined stromal compartments result in uniformly high lactate uptake with limited spatial heterogeneity.

These findings suggest that metastatic tumor cells exploit a pre-existing lactate-rich environment in the hepatic parenchyma, which may be further supported by lactate-producing CAFs in the surrounding stroma. This scenario echoes the “reverse Warburg effect,” where stromal glycolysis provides metabolic support to adjacent cancer cells^3^.

However, the reaction-level resolution of spFBA reveals that lactate metabolism in CRC liver metastases diverges markedly from the canonical model. In the classical reverse Warburg effect, lactate is oxidized through the mitochondrial TCA cycle via LDH-mediated conversion to pyruvate, followed by PDH entry into the TCA and subsequent respiration. In our case, although tumor cells do exhibit elevated respiratory chain activity, the fluxes show that lactate-derived pyruvate is not primarily used to fuel oxidative metabolism. Instead, pyruvate transport is enriched from mitochondria to the cytosol, suggesting that cytosolic biosynthetic pathways are the primary destination for lactate-derived carbons.

**Fig. 6:**
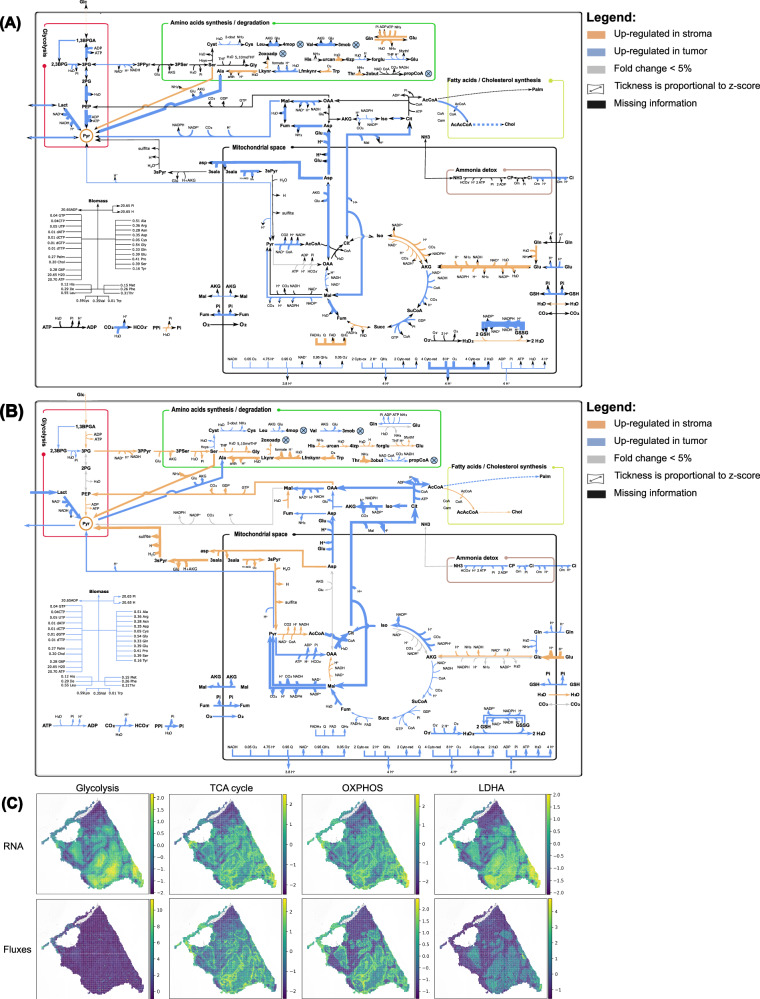
Stroma vs Tumor. **A** Differences in Reaction Activity Scores (RAS) between stroma and tumor spots in the CRC liver metastasis sample LM4. **B** Differences in Flux Enrichment Scores (FES) in the same sample. For both (**A**) and (**B**), reactions were tested using the Kolmogorov-Smirnov test (*α* = 0.05) and were considered directionally regulated only if they displayed an absolute fold change above 5%, as described in the legend. The full metabolic maps are provided in Fig. S15. **C** Spatial distribution of metabolic pathway scores in the same sample, computed by averaging the expression levels of metabolic genes within predefined metabolic subsystems and, analogously, using the absolute values of FESs in each set.

To better characterize the metabolic differences between tumor and stromal regions, we generated a reaction-level map of statistically significant flux differences (Fig. 6). For comparison, a similar map based on RAS is shown alongside, allowing a direct evaluation of the added value of flux-level modeling.

The FES-based differential map not only quantifies the magnitude of flux differences but also preserves directionality, enabling mechanistic insight. It shows that lactate is indeed converted to pyruvate, but instead of entering the TCA cycle through PDH, pyruvate is used for transamination with glutamate to form alanine and *α*-ketoglutarate (*α*KG). The resulting *α*KG is then imported into mitochondria in exchange for malate, via the *α*KG/malate antiporter. Inside the mitochondria, *α*KG sustains a non-canonical mode of TCA operation, supporting reductive carboxylation to export citrate for fatty acid synthesis. Despite bypassing the canonical TCA entry via PDH, this metabolic circuit still drives high activity through respiratory complex I, consistent with the elevated oxygen consumption predicted in tumor regions.

In the LM4 liver metastasis sample, the gene-expression-based GSEA highlights broad inflammatory and stress-related programs, indicating a highly immune-reactive tumor compartment (Fig. S14). However, this co-occurrence with lactate consumption does not establish a functional or spatial link between the two, which would require targeted investigation.

Collectively, these findings demonstrate that CRC liver metastases utilize lactate extensively, but not through conventional oxidative pathways. Instead, they reroute lactate-derived carbons via transaminase reactions and reductive TCA cycling to support biosynthesis and proliferation, revealing a non-canonical but highly effective mode of metabolic rewiring.

### Spatial distribution of metabolite exchange highlights tumor-stroma interface activity

The statistical analyses comparing tumor and stromal regions revealed significant differences in some metabolic fluxes. However, for certain reactions such as biomass production or oxygen consumption, these differences appeared less pronounced. This limited contrast is likely driven by intratumoral heterogeneity, rather than a uniform metabolic profile across the tumor region.

Spatial mapping through spFBA uncovers this heterogeneity, demonstrating that metabolic activity is not evenly distributed within tumor regions. As noticeable in Fig. 6 and better highlighted in Fig. S16, glutamate exchange exemplifies this phenomenon. While overall glutamate consumption is lower in tumor regions compared to the stroma, spatial visualization reveals distinct metabolic behaviors at the tumor-stroma interface. Black arrows indicate that glutamate production is concentrated at the tumor periphery, where cells are in direct contact with stromal regions. In contrast, cells within the tumor core predominantly consume glutamate, albeit at a lower rate than stromal cells.

Notably, this pattern is absent in primary tumor regions that lack stromal interaction. In primary tumors, glutamate is consistently consumed across the entire region, and no peripheral production is observed. This suggests that glutamate production may be specifically induced by the tumor’s interaction with stromal cells, highlighting a possible metabolic adaptation at the invasive front. Worth of note, according to the literature, glutamate exchange could be related to acquired drug resistance or epithelial-mesenchymal transition^42^.

Interestingly, glutamate secretion at the tumor-stroma interface coincides with regions of elevated blood vessel marker expression (Fig. 5C), suggesting that vascularization may support metabolite exchange or increased metabolic activity at the interface.

These findings underscore the advantage of spatial data, as single-cell analyses alone would only capture aggregate differences in metabolite consumption or production. By visualizing where metabolite exchange occurs, spFBA, coupled with high resolution provided by stereo-seq data, provides a more nuanced understanding of intratumoral metabolic heterogeneity and identifies regions at the tumor-stroma interface as potential hotspots of metabolic rewiring.

### spFBA predictions are coherent and biologically grounded across tumor datasets

At first glance, the extensive lactate consumption predicted in tumor cells from CRC liver metastases appeared unexpected. Tumor metabolism is classically associated with lactate secretion and glucose dependency. Yet, spFBA consistently predicted robust lactate uptake in tumor regions, particularly in liver metastases, while stromal regions exhibited low uptake or even active secretion in some areas.

Notably, this pattern does not appear in the renal cancer dataset, where no significant lactate uptake is predicted, despite the use of the same model structure and comparable extracellular conditions. This observation suggests that the lactate uptake seen in CRC is not an artifact of model topology, but rather emerges from the spatial features of the transcriptomic data. Nevertheless, given that spFBA predicts simulated fluxes, it is reasonable to question whether this behavior could result from a modeling illusion.

To address this, we compared flux predictions with RASs, which are directly computed from gene expression and independent of metabolic modeling. Focusing on the CRC liver metastasis sample (LM4), we quantified the percentage of reactions that showed both a *p*-value lower than 0.05 and an absolute fold change greater than 0.2 in both datasets. Among these, 74% exhibited concordant fold change direction between RAS and flux predictions. This substantial agreement suggests that transcriptional regulation, as captured by RAS, plays a major role in driving the observed metabolic changes, reinforcing the consistency between gene expression-based activity scores and flux reprogramming. As shown in Fig. 6, reactions associated with genes exhibit strong agreement between tumor-stroma flux differences and the corresponding RAS variations. Moreover, spFBA provides directional information that adds interpretive value beyond transcript-based activity alone. Multiple examples illustrate this convergence: the conversion of serine to pyruvate is upregulated in stroma across both layers; the pyruvate-to-lactate reaction is downregulated in stroma, as well as citrate export (via antiport with malate). Importantly, spFBA also generates predictions for reactions without direct gene associations, such as mitochondrial oxygen uptake or biomass production, which are absent from the RAS layer but clearly resolved in the FES maps.

Biomass production, in particular, is predicted in a manner that aligns with biological expectations: tumor regions consistently exhibit higher biomass fluxes than stromal regions, consistent with their known proliferative behavior. This not only demonstrates the added value of flux-level modeling but also supports the overall reliability of spFBA predictions.

Beyond confirming patterns visible in transcriptomic data, spFBA also reveals activity where gene expression is silent. For instance, the 3-phosphoglycerate (3PG) to serine pathway is upregulated in the stroma, despite missing gene annotations for one or more of its components. Such predictions can guide hypotheses in cases where transcriptomic coverage is incomplete.

spFBA also resolves inconsistencies that commonly arise within linear pathways. In several cases, consecutive enzymes display heterogeneous or even contradictory RAS values. For instance, in the synthesis of the Alanine (Ala) from Tryptophan (Trp), some steps appear upregulated in the stroma while others appear upregulated in tumors at the transcript level (Fig. 6A). Because spFBA enforces stoichiometric coupling and steady-state mass balance, reactions arranged in series cannot carry discordant fluxes; instead, the entire pathway must adopt a coherent rate. Accordingly, the corresponding flux map (Fig. 6B) shows a smooth, uniform up-regulated flux profile for the stroma condition.

Occasionally, RAS and flux predictions diverge. A notable example is isocitrate dehydrogenase, which appears upregulated at the transcript level but shows reduced flux. Such discrepancies are biologically plausible: enzyme expression may be up-regulated in response to low substrate availability, even if actual flux through the reaction decreases. The flux through this reaction in tumor is indeed sustained by the AKG derived from pyruvate.

To evaluate this correspondence in a spatially resolved manner, we computed and visualized pathway-level activity scores based on both RAS and FES data (Fig. S11C). Among the pathways analyzed—glycolysis, TCA cycle, respiratory chain, and lactate dehydrogenase—we observed strong agreement in most cases, with the notable exception of glycolysis. While transcript-based scores revealed substantial heterogeneity among glycolysis-related genes, the predicted fluxes appeared spatially homogeneous, likely reflecting stoichiometric and energetic constraints. In contrast, other pathways, such as the TCA cycle, lactate dehydrogenase, and the respiratory chain, retained high spatial heterogeneity in both layers.

**Fig. 7:**
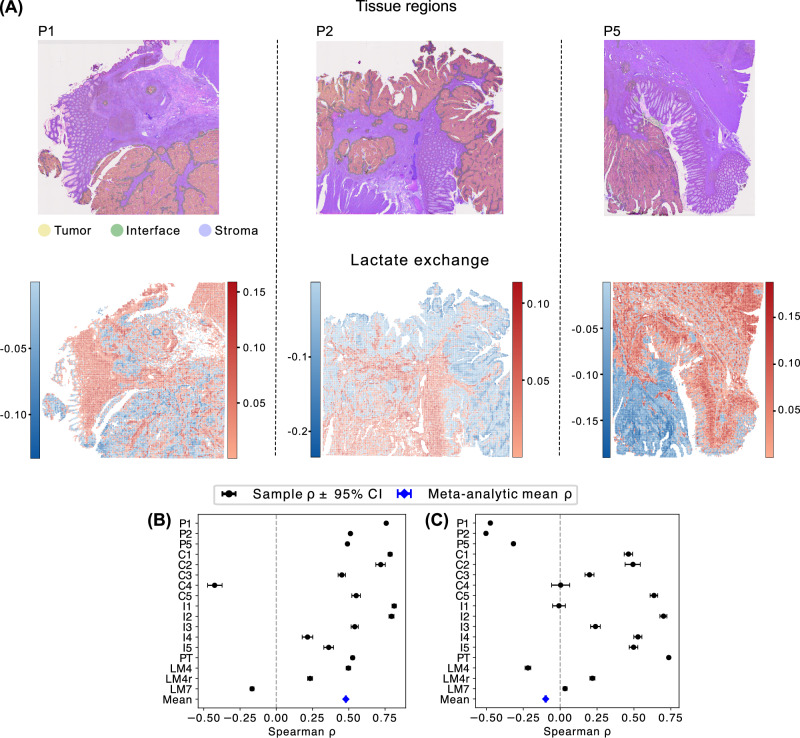
spFBA results across datasets. **A** Columns correspond to samples P1, P2, and P5, from left to right. **Top-row** shows tissue regions annotated as Tumor (yellow), Interface (green), and Stroma (purple) overlaid on the histopathological image. **Bottom-row** shows spatial distribution of Lactate consumption (blue) and production (red). For completeness, further information on these samples is reported in Supplementary Fig. S18. **B** Spearman correlations between proliferation markers and Biomass FES across all samples. Individual points denote sample-specific estimates with 95% confidence intervals, and the diamond indicates the inverse-variance weighted meta-analytic mean *ρ*. **C** Spearman correlation between blood-vessel markers and *O*_2_ consumption, using the same visualization schema.

To further exclude the possibility that lactate consumption in CRC metastases is a modeling artifact, we tested spFBA on an independent CRC dataset generated using a different ST technology (i.e., 10x Visum HD)^43^. Despite the differences in platform, resolution, and preprocessing, the same pattern emerged: tumor regions consistently displayed strong lactate uptake, whereas stromal areas exhibited minimal uptake or even secretion (Fig. 7A).

This convergence across independent datasets strengthens the robustness of our finding. In both CRC datasets, despite their distinct technical origins, tumor regions preferentially consume lactate through non-canonical metabolic routes, whereas this behavior is absent in renal cancer samples. Together, these observations indicate that lactate consumption is likely a reproducible and CRC-specific metabolic feature rather than a consequence of model assumptions or dataset-specific biases.

Beyond tumor-stroma comparisons in individual sections, we next sought to assess - across the full set of CRC and renal samples - whether spFBA predictions covary with spatial features that are mechanistically expected to influence metabolic activity. To this end, we performed cross-sample validation analyses designed to test whether predicted biomass flux covaries with non-metabolic proliferation markers. We remark that, in contrast to classical FBA formulations, spFBA does not optimize or impose biomass flux. Each spatial unit is free to carry zero, low, or high biomass flux depending solely on the expression profile of metabolic genes. Consequently, spatial variations in biomass production arise emergently from the data-driven feasible space, rather than from any assumption that cells must grow or prioritize proliferation.

Figure 7B shows that the correlation between biomass FES and proliferation score is highly positive in most samples, with an average Spearman correlation of about 0.5. This result reinforces the ability of spFBA to generate biologically coherent and spatially resolved predictions of metabolic activity by integrating gene expression with physiological context.

The robustness of spFBA predictions was further supported by the observed overlap between predicted hypoxic regions and spatial markers of blood vessels in the ccRCC samples. We sought to quantitatively assess the relationship between oxygen consumption rates and vascularization across all samples. However, this analysis is complicated by the fact that spots enriched in blood vessels are likely to include a high proportion of blood cells, whose metabolism may confound the signal from surrounding cancer cells. Moreover, a perfect correlation between oxygen consumption and vascularization should not be expected: oxygen usage depends on the intrinsic metabolic activity of cells, not solely on local oxygen availability. Indeed, the scatter plots in Fig. S17 clearly indicate a non-linear relationship between the two variables. To explore this further, we computed Spearman correlation coefficients (Fig. 7C). Interestingly, most ccRCC samples, profiled using Visium, display a positive correlation between oxygen consumption and vascularization. In contrast, CRC samples profiled with Stereo-seq show negligible correlation, while Visium HD datasets even exhibit moderately negative correlations. These results suggest that spatial resolution affects the observed correlation, potentially due to differences in the relative abundance of blood cells captured within each spot.

Finally, it is important to note that all spFBA-predicted fluxes are stoichiometrically balanced and mass-conserving. The underlying CB model enforces steady-state conditions, elemental and cofactor balance, and ensures full redox coupling. As a result, all predicted flux configurations remain biochemically feasible and consistent with known conservation laws.

Taken together, these cross-dataset associations provide an orthogonal validation layer confirming the biological plausibility and interpretability of the inferred fluxes.

## Discussion

Our spatially resolved metabolic analysis of colorectal and renal tumors uncovered multiple, distinct metabolic phenotypes associated with lactate handling. In CRC liver metastases, tumor cells consistently consumed lactate, but did so through a non-canonical pathway: instead of oxidizing lactate-derived pyruvate via the TCA cycle, they redirected it toward biosynthesis via transamination and mitochondrial citrate export. This flux pattern, which we define as a pseudo-reverse Warburg effect, suggests that lactate serves as an anabolic substrate rather than as a respiratory fuel. Stromal regions, by contrast, showed low lactate uptake and occasional secretion, suggesting a possible source-sink dynamic within the tumor microenvironment.

In renal cancer, tumor cells predominantly secreted lactate, consistent with their highly glycolytic phenotype. However, spFBA revealed further spatial diversity within this overall pattern: some tumor regions secreted lactate despite active oxygen uptake, consistent with a “hybrid” Warburg phenotype; others showed classical anaerobic glycolysis with reduced respiratory activity. This spatial stratification of the Warburg effect illustrates how local oxygen availability and vascularization can shape metabolic strategy within the same tumor.

These findings were enabled by spFBA, a computational framework that integrates ST with CB modeling and flux sampling to infer directionally resolved reaction-level activity. Unlike enrichment methods (Fig. S14), spFBA provides predictions for reactions lacking gene annotations and captures flux directionality, allowing the detection of metabolic programs invisible to transcript-based metrics alone.

The resulting FES maps can be used not only for visualization and interpretation, but also as input for downstream spatial analyses. For instance, one can correlate fluxes with distance from the tumor-stroma interface, search for complementary exchange fluxes across compartments, or identify regional markers of nutrient dependency. The CRC dataset we provide, together with its matched flux predictions, constitutes a resource for such investigations.

While spFBA does not quantify absolute flux values, its predictions are mass-balanced, stoichiometrically feasible, and mechanistically interpretable. Across datasets and tissue types, it captured known cancer metabolic hallmarks—including elevated glycolysis, biomass production, and lactate secretion—as well as spatially organized, tissue-specific metabolic strategies.

Rather than providing definitive validation of a metabolic mechanism, spFBA is intended to generate structured, data-driven hypotheses that can inform and guide future experimental investigations.

While spFBA focuses on metabolic differences, integrating analyses of differentially expressed genes and pathway enrichment could provide deeper insights into both the consequences of metabolic rewiring and its potential regulatory drivers. To this aim, a recently proposed approach combining flux sampling-based predictions with machine learning algorithms could be applied in future work to associate metabolic features predicted by spFBA with non-metabolic biological processes^44^.

In this study, we applied spFBA to ST data from a total of 17 samples from 11 cancer patients, including 4 CRC primary tumors, 3 liver metastases, and 10 ccRCC cases. While this sample size is not sufficient to draw universal conclusions about tumor metabolism, the consistency of our findings across independent datasets suggests that spFBA can robustly identify spatially resolved metabolic patterns. Given that spFBA can be applied to any sequencing-based spatial dataset, and that such datasets are rapidly increasing in number, resolution, and anatomical diversity, this framework can be systematically employed to investigate context-specific metabolic features across a broad range of biological questions.

Moreover, we are currently developing a scalable implementation of spFBA tailored to imaging-based platforms such as CosMx, which generate datasets at single-cell resolution and on a much larger scale^45^. The analysis of these datasets will significantly enhance the characterization of spatial metabolism and cell-cell interactions.

## Methods

### ccRCC dataset

We analyzed a publicly available ST dataset^24^, where kidney samples from 7 patients with ccRCC were sequenced using the 10x Visium Genomics protocol.

The patients underwent surgical resection of radiologically diagnosed, treatment-naive renal tumors. Most of the patients were in significantly advanced stages of the disease, we refer the reader to^24^ for further details. Among the 16 samples available, we kept 5 samples from the tumor core and 5 from the interface between primary tumor and healthy tissue, with higher quality considering metadata annotations provided by the original authors. See Table 1.

**Table 1:** Dataset metadata

Sample name	Sample ID	Patient ID	Region	Spot area [μm]	Spot #	Unique genes	Mean counts per spot	Mean genes per spot
LM4	A02991A2	SC087	Liver met.	625	9539	24,673	3526.04 ± 1290.24	1630.53 ± 512.48
LM7	C03445C6	SC087	Liver met.	625	20,729	25,425	2569.98 ± 812.26	1348.27 ± 362.47
PT	C03445G5	SC087	Colon primary tum.	625	14,956	25,688	5592.64 ± 2137.28	2224.24 ± 562.71
LM4r	A03389C5	SC087	Liver met.	625	11,919	24,974	4912.33 ± 1389.13	1941.12 ± 479.57
C1	6800STDY12499410	PD47171	Kidney - Tum. Core	2376	2888	22,317	5821.02 ± 3532.71	2334.16 ± 1002.04
C5	6800STDY12499413	PD45816	Kidney - Tum. Core	2376	2102	19,735	2776.64 ± 1891.10	1193.50 ± 549.47
C2	6800STDY12499407	PD43948	Kidney - Tum. Core	2376	832	19,822	10290.89 ± 5756.34	3065.35 ± 1101.38
C4	6800STDY12499412	PD45814	Kidney - Tum. Core	2376	1026	21,488	9650.70 ± 4667.24	3324.03 ± 1184.36
C3	6800STDY12499408	PD47512	Kidney - Tum. Core	2376	3800	22,184	3323.62 ± 2214.55	1778.00 ± 779.10
I4	6800STDY12499502	PD45814	Kidney - Tum. Interface	2376	2657	20,693	2215.16 ± 1544.44	1050.37 ± 510.51
I2	6800STDY12499506	PD45816	Kidney - Tum. Interface	2376	1777	21,399	5384.21 ± 2836.88	2242.85 ± 873.76
I5	6800STDY12499508	PD47465	Kidney - Tum. Interface	2376	2609	21,840	3565.95 ± 2075.09	1520.86 ± 650.56
I3	6800STDY12499406	PD43824	Kidney - Tum. Interface	2376	3153	22,149	2479.87 ± 2041.36	1191.62 ± 657.54
I1	6800STDY12499411	PD47171	Kidney - Tum. Interface	2376	2048	23,116	8371.10 ± 5489.16	2993.72 ± 1393.52
P1	-	P1	Colon primary tum.	1024	26,687	18,054	3859.93 ± 2794.79	2020.16 ± 110.35
P2	-	P2	Colon primary tum.	1024	31,414	18,072	8431.28 ± 5748.10	3654.41 ± 858.91
P5	-	P5	Colon primary tum.	1024	31,773	18,058	7449.18 ± 7151.15	2920.52 ± 890.47

Additional information regarding patient, sample, and quality metrics is reported for each dataset.

### CRC in-house dataset

We included in this study a primary tumor biopsy (PT) and two liver metastases (LM4 and LM7). Biopsies have been collected in the Department of Oncology, University of Leuven, from a single patient (*SC087*). For LM4, we generated a technical replicate (LM4r). Slices from PT and LM7 were smaller than LM4, so it was possible to put two consecutive slices on the same stereo-seq chip.

### CRC public dataset

We processed publicly available datasets generated with 10x VisiumHD platform. FFPE blocks were collected from three patients with colon adenocarcinoma. In particular, we considered samples labeled as P1, P2, and P5.^43^. Raw counts data were downloaded from 10x Genomics website, while the metadata with tissue annotations were obtained from the GitHub repository reported in the original article. Prior to process the raw count matrices, as explained below, 8*μm* spots were aggregated in 32 × 32 μm bins, in order to reduce data sparsity and dataset size. We then removed bins with less than 100 detected genes. Moreover, original authors annotated each spot in one of the following tissue regions Tumor, Stroma, and *50 micron* (i.e., the interface between the other two regions). We annotated the aggregated bins considering the proportion of the original label among the 16 spots. If the proportion of Tumor or Stroma was higher than 0.5, we kept that annotation, otherwise we annotated the given bin as Interface.

### Donors and sample collection

Biopsies from a patient diagnosed with CRC and liver metastasis were received fresh after written informed consent, according to the Declaration of Helsinki. Approval by the medical ethics commission of the KU Leuven University (S50887) was obtained, in accordance with the local ethical guidelines. Samples were collected from a 72-year-old male patient. (*SC087*). Before surgery, patient received a combination of immunotherapy (i.e., Cetuximab) and chemotherapy (i.e., Levofolinezuur, Oxaliplatine plus Fluorouracil) treatment.

### Histological analysis

To assess the morphological features, tissue structure, and preservation, we performed Hematoxylin and Eosin (H&E) staining of each tissue block. The fresh frozen samples were sectioned at a thickness of 10 μm and fixed with methanol. Afterward, sections were incubated with 500 μl of isopropanol for 1 min. Slides were air-dried for 5-10 min, followed by incubation with hematoxylin. Afterward, the slides were washed by immersion in water and air-dried. Subsequently, 1 ml of bluing buffer was applied and washed. Eosin mix was then added to stain the cytoplasmic components and extracellular matrix. After washing, the stained sections were imaged in a microscope using a BF-Epi channel, 4X and 10X objective lenses, with stitching function.

### Stereo-seq tissue optimization

Tissue optimization was performed using the Stereo-seq Permeabilization kit (Cat. No. 111KP118) and Stereo-seq chip set P (Cat. No. 110CP118) according to the manufacturer’s protocol (Stereo-seq permeabilization set user manual, Ver A1) to define the optimal permeabilization time for the tissue samples we specifically wanted to analyze. Briefly, 10 μm tissue sections were prepared from the primary tumor and liver metastasis cryo-blocks and placed on 2 permeabilization slides containing 4 chips each. The tissue layer was thawed to attach it to the surface of the chips. After drying the tissue at 37 ^∘^C, the slides were then dipped into pre-chilled 100% methanol at −20 ^∘^C and incubated for 30 ^∘^C minutes to fix the tissue. Post fixation, the tissue permeabilization test was performed on these chips by permeabilizing the tissue at 4 different time points (6, 12, 18, and 24 min). Afterward, reverse transcription was carried out at 42 ^∘^C for 1 h in dark, followed by tissue removal at 55 ^∘^C for 1 h. Fluorescence imaging was performed in the TRITC channel with 10X objective, following the imaging guidelines provided by the manufacturer (Guidebook for Image QC and microscope assessment and imaging, Ver A5). The optimal permeabilization time was assessed based on the strongest fluorescence signal with the lowest signal diffusion (crispness of the RNA footprint). We found the optimal permeabilization time for both the primary colon tumor and the liver metastasis to be between 12 and 18 min, and, thus, 15 min was used for the ST assay.

### Stereo-seq spatial transcriptomics assay

The spatial transcriptomics analysis was performed using the Stereo-seq Transcriptomics kit (Cat. No. 111ST114) according to the manufacturer’s protocol (Stereo-seq Transcriptomics set user manual, Ver A2). Briefly, cryosectioning, tissue mounting, and fixation were performed exactly as previously described in the protocol for tissue optimization. Next, each fixed tissue section was stained with Qbit ssDNA reagent. Fluorescence imaging of the single-stranded DNA staining was performed in the FITC channel with a 10X objective, following the imaging guidelines provided by the manufacturer (Guidebook for Image QC and microscope assessment and imaging, Ver A5). Prior to permeabilization, the ssDNA-stained image was also subjected to QC analysis using the imageQC software as recommended by the manufacturer. Tissues were then permeabilized for 15 min, as estimated in the tissue permeabilization test.

After washing the chip, reverse transcription was performed at 42 for 3 h. The tissue was then digested and removed from the chip, and cDNA was released, collected, and purified following the manufacturer’s recommendation. After quality assessment using a bioanalyzer (Agilent), sequencing library preparation was performed using transposase-assisted tagmentation reaction. Indexed PCR and library purification were performed to prepare the final sequencing library as per manufacturer’s recommendations. Final Stereo-seq libraries were sequenced on MGI/BGI sequencing platforms (DNBSEQTM T7) at the MGI Latvia sequencing facility.

### Stereo-seq sequencing processing

We processed each FASTQ file obtained from each sample using the SAW pipeline v.7.1.1^46^. The pipeline consists of two main steps, which were performed for each sample as follows: (1) *Alignment and Counting*: We used Homo_sapiens.GRCh38.dna.primary_assembly.fa, Ensembl release 111, as the reference genome and the corresponding GTF annotation file (Homo_sapiens.GRCh38.111.gtf). The sample-related chip mask file, which includes the spatial coordinates, was part of the transcriptomic kit provided by STOmics. We reported the sample IDs in Table 1. (2) *Image Registration*: The fluorescence image taken during the ST *Assay* was aligned to the count matrix to define the tissue area, filtering out DNA nano-balls that were not under the tissue from the count matrix.

The pipeline outputs the registered image and a table (.gem/.gef) that includes, for each unique combination of gene ID and spatial coordinates (x, y), the number of molecular identifier (MID) counts. This table was further processed with Stereopy v1.3.0^47^ to define the final count matrix by aggregating spatial coordinates into spots of 50 × 50 DNA nano-balls. Therefore, the dimension of each spot is 25 × 25 μm^2^. The obtained count matrix (spot × gene) is stored in a Scanpy object for downstream analyses.

### ST read counts preprocessing

We applied the same preprocessing steps for both Visium and Stereo-seq datasets using Scanpy v1.9.6^48^. In particular, the expression profile in each spot was normalized by library size (pp.normalize_total(adata=adata, target_sum=1e4)) and log-transformed (pp.log1p(adata)) as usually done in single-cell data analysis^49^.

Subsequently, we ran PCA (tl.pca(adata, svd_solver=‘arpack’)) and selected the first 30 PCs as input for k-nearest-neighbor graph (kNN) construction (pp.neighbors(adata, n_neighbors=10, n_pcs=30)).

Next, we clustered kNN graph nodes using the Leiden approach (tl.leiden(adata)), selecting a resolution specific to each dataset. We explored the data quality by plotting the distribution of detected genes and total counts in each cluster via violin plots. If needed, we removed clusters with particularly low numbers of detected genes and counts. The resulting datasets have the dimensions reported in Table 1.

Finally, to reduce data sparsity, we applied MAGIC imputation^50^ with default parameters, before processing the datasets with spFBA.

### Metabolic model

spFBA theoretically accepts any metabolic network model. However, we were more confident using the manually curated ENGRO2 core network model^14^ of the human central carbon and essential amino acids metabolism. Specifically, we used the recently published updated version ENGRO2.2^44^, which comprises 395 metabolites, 469 reactions, and 498 genes. Among the 469 reactions, 351 are associated with GPR rules, enabling robust integration of transcriptomic data into the model.

The biomass pseudo-reaction corresponds with the biomass reaction of the Recon3D model, in terms of the set of metabolites considered and corresponding stoichiometric coefficients. We simulated a growth medium condition in which exogenous metabolites are abundantly available, or an open medium. Exogenous metabolites in the ENGRO2 network include glucose, lactate, oxygen, water, hydrogen, folic acid, palmitate, and all essential and non-essential amino acids.

### Reaction activity scores

We computed a RAS for each reaction and spot by substituting the mRNA abundances into the corresponding Gene-Protein-Reaction (GPR) rule, as done in ref. ^51^. To solve the logical expressions, the minimum transcript value was taken when multiple genes are joined by an AND operator, and the sum of their values was taken when multiple genes are joined by an OR operator. In the case of GPRs combining both operators AND and OR, we respected their standard precedence.

Once a RAS was computed for each reaction in the metabolic model, we obtained a table of dimensions Spots x Reactions, containing the RAS values.

### Flux variability analysis

To determine the extreme points of the feasible space, that is, the range of possible fluxes that satisfy the mass balance and the medium constraints, we used FVA^52–54^. FVA is a CB modeling technique aimed at determining the maximal and minimal possible flux through any reaction of the model. FVA solves the following two linear programming optimization problems (one for minimization and one for maximization) for each flux *v*_*j*_ of interest, with *j* = 1, …, *R*:

1 $$\begin{array}{l}\max /\min \,{v}_{j}\\ S\cdot \vec{v}=\vec{0}\\ \vec{{v}_{L}}\le \vec{v}\le \vec{{v}_{U}}\end{array}$$

where *S* is the stoichiometric matrix provided by a metabolic network model and $\vec{v}$ is the vector representing the flux of each reaction, $S\cdot \vec{v}=0$ represents the steady state assumption. The vectors $\vec{{v}_{L}}$ and $\vec{{v}_{U}}$ represent the predefined lower and upper flux boundaries used to mimic as closely as possible the biological process in the analysis and the availability of nutrients in the medium. For each reaction *j*, the solutions of the two optimisation problems yield the minimal and maximal feasible flux values, denoted ${F}_{j}^{l}$ and ${F}_{j}^{u}$, respectively. These values represent the extremal fluxes allowed by the network, and therefore can be more restrictive than the predefined bounds of any reaction. We assumed a rich open medium where all exogenous metabolites are made available in an unlimited quantity, which is 1000 in the model.

### Spot-relative flux constraints

After the RAS and FVA computation, specific constraints on internal fluxes of the network are built following an approach adapted from^14,15^. For each reaction *j* = 1, …, *R* and spot *s* = 1, …, *S*, an upper bound ${U}_{j}^{s}$ and a lower bound ${L}_{j}^{s}$ to the flux capacity are defined, based on the following formulas:

2 $${U}_{j}^{s}={F}_{j}^{u}\times \frac{{RAS}_{j}^{s}}{{\max}_{s}{RAS}_{j}^{s}},$$

3 $${L}_{j}^{s}={F}_{j}^{l}\times \frac{{RAS}_{j}^{s}}{{\max }_{s}{RAS}_{j}^{s}},$$

where ${F}_{j}^{u}$ and ${F}_{j}^{l}$ represent the maximum and the minimum flux that reaction *j* might carry, obtained by FVA, and ${RAS}_{j}^{s}$ is the RAS value for spot *s* and reaction *j*. These constraints are used to map the transcriptomics data into spot lattice, with a one-to-one correspondence between the single spot transcriptomics profile and the corresponding network of each spot. Therefore, each sub-network has specific constraints derived from the transcriptomics and defined in Eqs. (2) and (3).

### Parsimonious FBA

Given the new set of spot-relative constraints for each spot, we used Eq. (1) to determine the flux distribution that optimizes metabolic growth (i.e., the flux of the biomass synthesis pseudo-reaction) for each spot. Because the optimization problem might have alternative solutions, to select a single flux distribution, we employed parsimonious FBA (pFBA). pFBA operates in two optimization steps. First, it determines the maximal value of the biomass. Then, it minimizes the total sum of reaction fluxes while maintaining the optimal value of the primary objective^17^.

### Corner-based sampling

To analyze each spot-relative metabolic network without assuming an objective function, spFBA relies on flux sampling of the feasible region to generate a sequence of feasible solutions that satisfy the network constraints. Flux sampling provides information not only on the range of feasible flux solutions, like FVA, but also on their probability.

In this study, we employed a Corner-based algorithm, namely CB_3_^19,55^, to sample the vertices of the feasible region by using different weighted objective functions each time as the objective function. A predefined number of samples is determined. At each iteration, a new objective function is set by randomly assigning weights in the range [−1, 1] and deciding whether the objective function will be maximized or minimized. To account for the different scales of the various reactions in the model, the weights are divided by the FVA maximum value for each specific reaction. More details of the implementation are reported in ref. ^19^. Such an algorithm captures flux distributions qualitatively and quantitatively more heterogeneous compared to classical Hit-and-Run strategies. In this work, we sampled 10000 points for each spot.

### Flux enrichment score (FES)

For each spot, we calculated a single FES using the following approach. For the pFBA method, we obtained the unique flux distribution by solving the corresponding optimization problem. We then normalized each reaction value by taking the maximum of the FVA if the flux value is positive, and the minimum of the FVA otherwise. This normalization allows us to assign a score between −1 and 1 for each reaction. In the case of the CB_3_ method, we computed the centroid from 1000 samples, which yields a feasible flux distribution based on the average of all sampled fluxes. We then applied the same normalization process as in the pFBA method. For each FES we also computed its 99% confidence interval.

### Spots clustering

For each layer of features, namely counts, RAS, and the two different types of fluxes (pFBA ad CB_3_), we first applied Principal Component Analysis (PCA) to reduce the dimensionality of the datasets. The neighborhood graph was computed on the PCA space to create a distance matrix, encoding the connectivity between spots based on Euclidean distance^56^. The Leiden community detection algorithm was then performed to identify the optimal communities representing clusters of well-connected spots within the neighborhood graph^57^.

To produce comparable clusters, we optimized the clustering parameters by maximizing the silhouette score^58^, by testing different parameter combinations with a grid-search strategy. The parameters optimized included the number of principal components, the number of neighbors when computing the neighborhood graph, and the resolution in the Leiden algorithm.

### Vascularization score

To evaluate vascularization across spatial spots, we computed a blood vessel gene expression score by averaging the normalized expression of six well-established endothelial markers: CD31 (PECAM1), ESAM, CD34, TEK (TIE2), VCAN, and CDH5 (VE-cadherin). These genes were selected based on prior studies that consistently report their specificity and reliability as endothelial markers in both physiological and pathological contexts^59–63^. The resulting per-spot vascularization score was used to assess the relationship between metabolic activity and vascular density via Spearman correlation with predicted *O*_2_ consumption rates.

### Proliferation score

To quantify proliferative activity across spatial spots, we calculated a proliferation gene expression score by averaging the normalized expression of four canonical cell-cycle markers: *MCM7*, *MCM3*, *PCNA*, and *MKI67*. These genes are widely used as indicators of active DNA replication or cell-cycle progression and, importantly, are not directly linked to metabolic pathways^64^. The resulting per-spot proliferation score was then correlated with predicted *Biomass* production rates using Spearman correlation to examine how regional proliferative demand relates to metabolic flux patterns.

### Pathway enrichment analysis

To evaluate pathway-level differences between Tumor and Stroma regions, we performed gene set enrichment analysis (GSEA)^65^ on ranked differential expression statistics. Signed gene-level effects were obtained using the rank_genes_groups function in Scanpy (v.1.9.8)^48^, specifying reference="Stroma" so that positive statistics indicate genes enriched in Tumor, whereas negative statistics indicate enrichment in Stroma. Differential expression was computed using logistic regression to obtain stable, signed effect sizes across all detected genes. Ensembl gene identifiers were mapped to HGNC gene symbols using the MyGene.info API (v.3.2.2). Only genes with valid symbol mappings were retained for downstream analysis. Genes were then ranked by their differential expression statistic and supplied to a preranked GSEA implementation (GSEApy v.1.1.2). Enrichment was performed against the MSigDB Hallmark collection using 1000 permutations and default size filters. Resulting normalized enrichment scores (NES) were interpreted such that positive NES correspond to pathways upregulated in Tumor and negative NES to pathways upregulated in Stroma.

## Supplementary information

Supplementary Information

## Data Availability

The raw and processed read counts datasets, including both ccRCC (10x Visium), CRC Primary Tumor, corresponding Liver Metastasis (Stereo-seq), and CRC (10x Visium HD) samples, are available in the following Zenodo repository: 10.5281/zenodo.13988865. The repository also includes the main output files. In particular, the FESs for all the samples.

## Code availability

The Python implementation of the spFBA computational pipeline, the code for processing the dataset and for generating the main figures are available in the following GitHub repository: https://github.com/CompBtBs/spFBA.git.
