# Supplementary material for: Spatial FBA reveals heterogeneous Warburg niches in renal tumors and lactate consumption in colorectal cancer
Source: NPJ Syst Biol Appl. 2026 Jan 27;12:32. doi: 10.1038/s41540-026-00654-x (PMC12948969; doi:10.1038/s41540-026-00654-x)
Supplement: Supplementary file 1 — Supplementary Information [file 41540_2026_654_MOESM1_ESM.pdf]

Supplementary Materials

Supplementary Figures

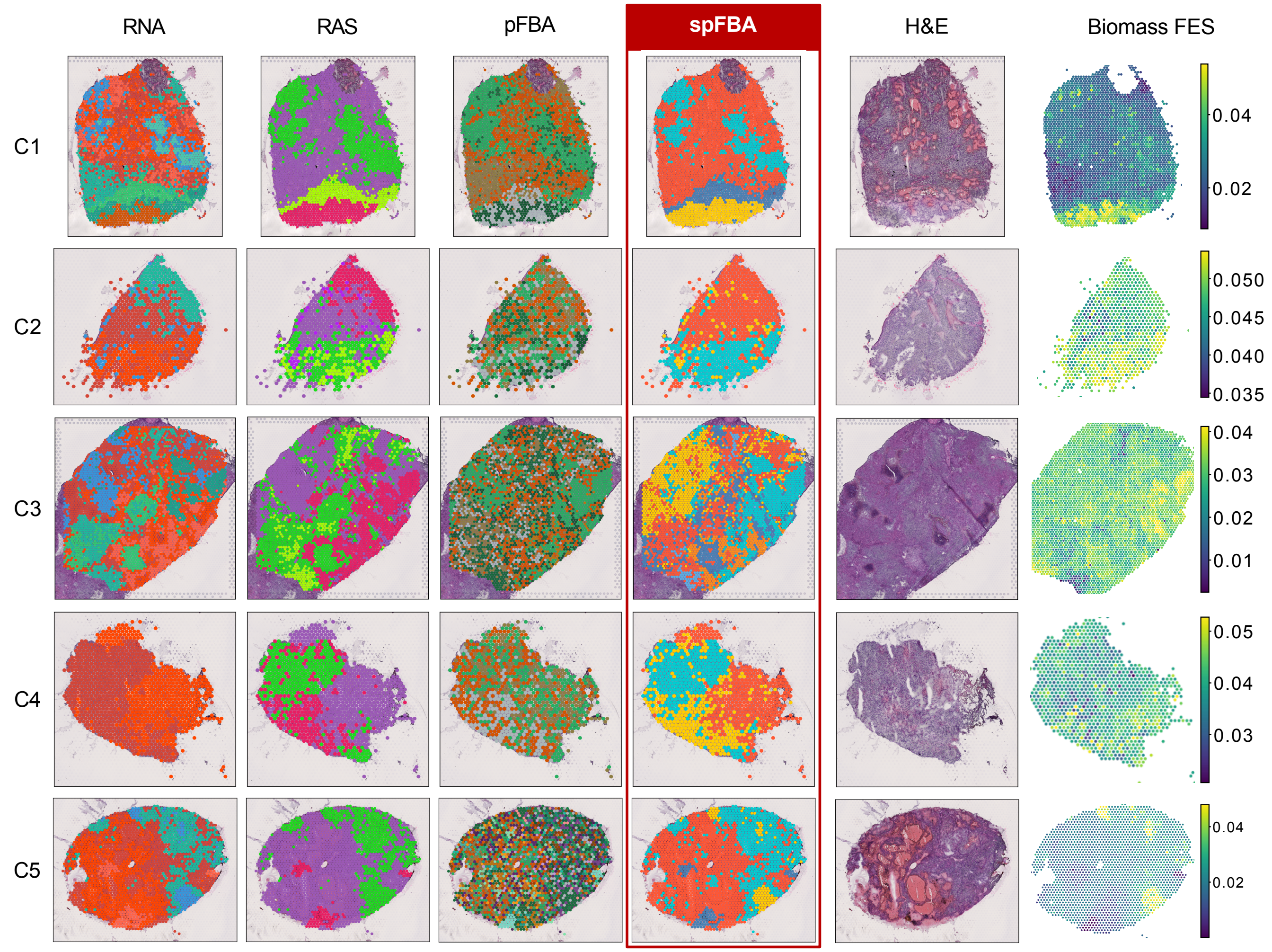

Figure S1: ccRCC tumor core samples. From left to right: H&E staining; clustering results obtained with different data layers: RNA (processed reads counts), RAS, FESs obtained with pFBA and spFBA - colors were arbitrarily assigned to clusters, thus same colored clusters in different plots are not directly comparable; Biomass FES obtained with spFBA

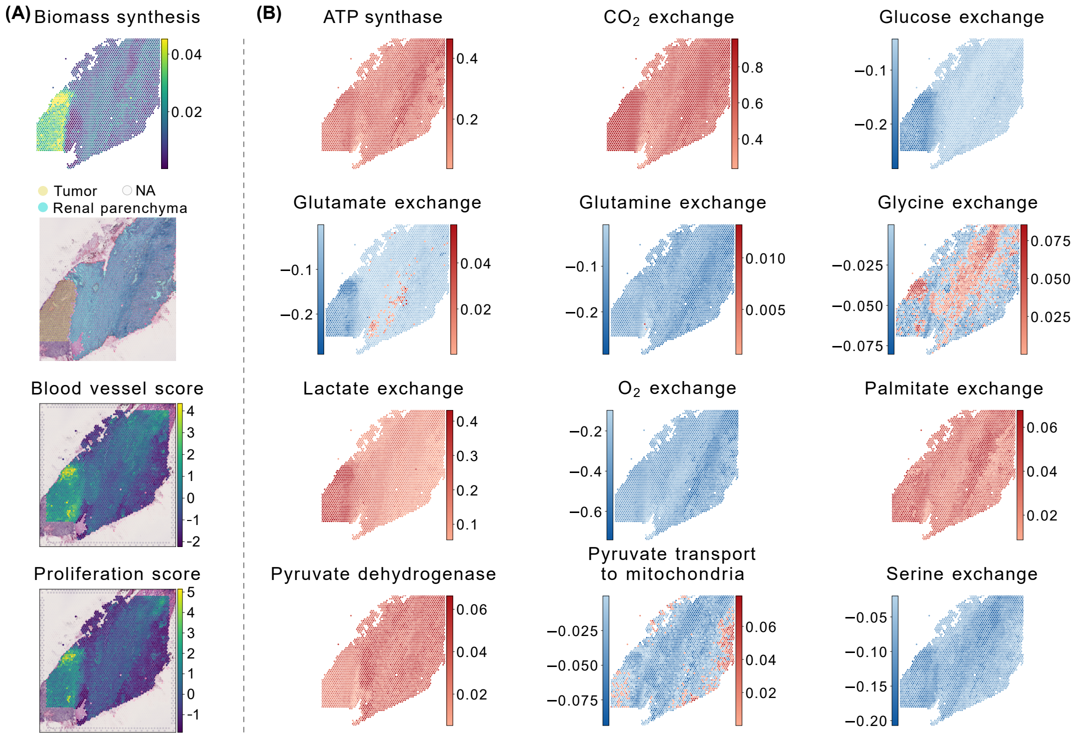

Figure S2: **ccRCC interface sample I3. (A)** Biomass FES, annotated H&E image, and blood vessel and proliferation scores derived from gene expression. **(B)** The FES of a set of reactions of interest (in alphabetic order) is visualized for the same sample. In the case of exchange reactions, negative values correspond to consumption of the metabolite, positive values to production.

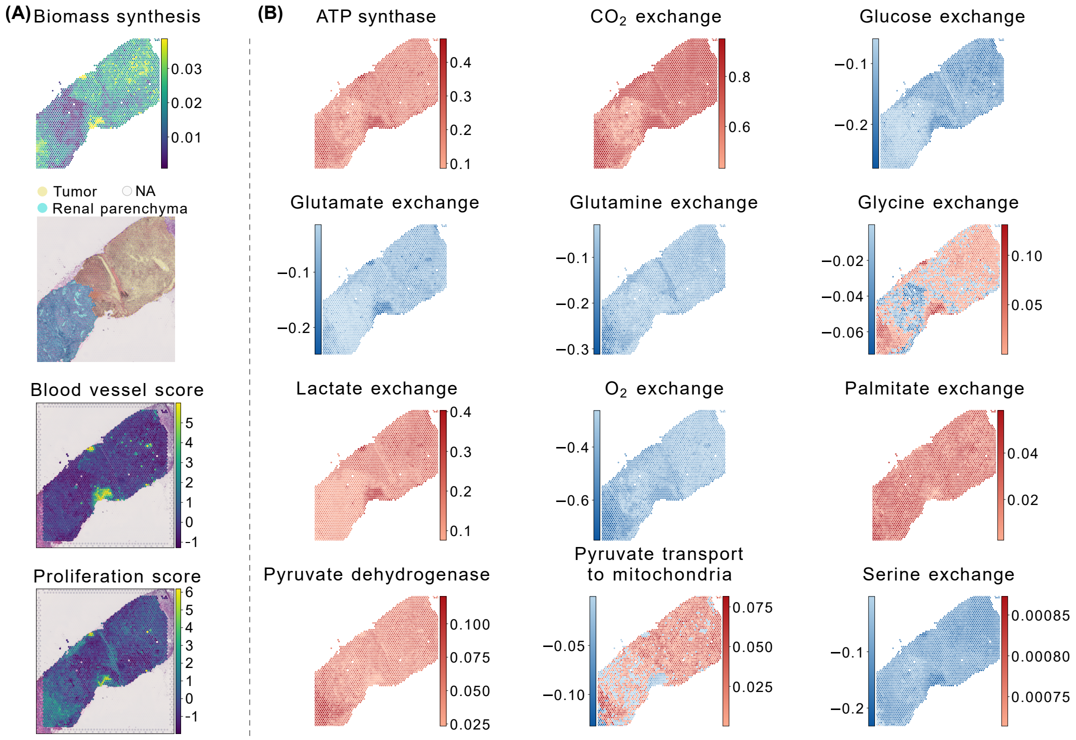

Figure S3: **ccRCC interface sample I4.** **(A)** Biomass FES, annotated H&E image, and blood vessel and proliferation scores derived from gene expression. **(B)** The FES of a set of reactions of interest (in alphabetic order) is visualized for the same sample. In the case of exchange reactions, negative values correspond to consumption of the metabolite, positive values to production.

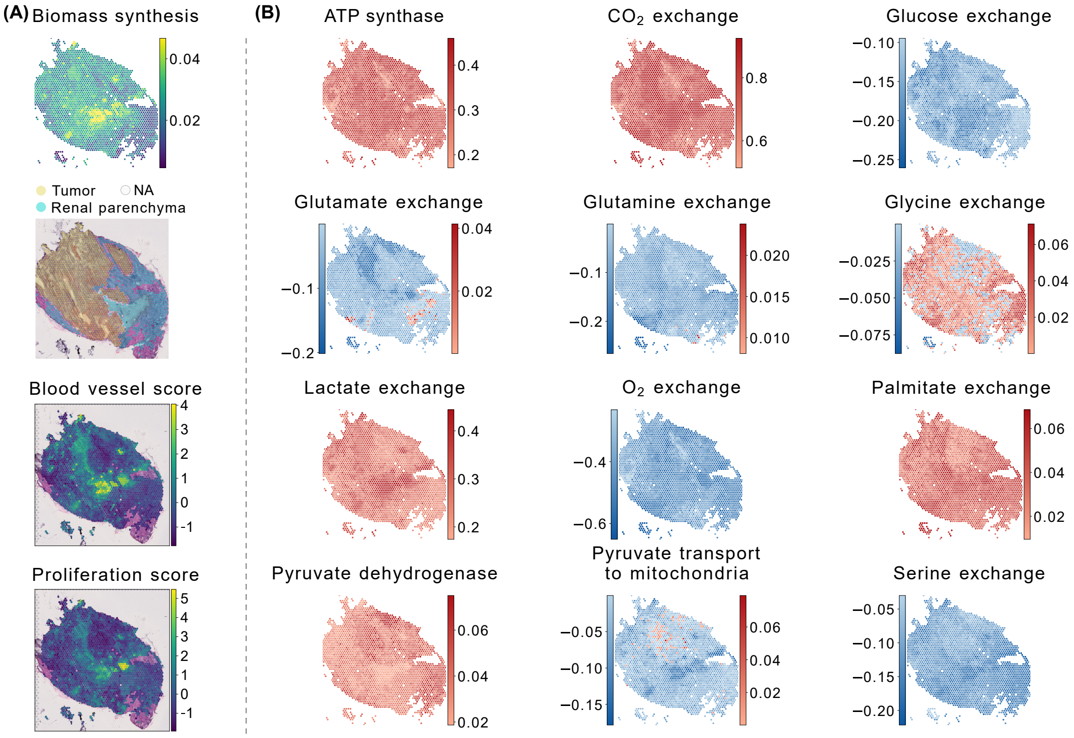

Figure S4: **ccRCC interface sample I5. (A)** Biomass FES, annotated H&E image, and blood vessel and proliferation scores derived from gene expression. **(B)** The FES of a set of reactions of interest (in alphabetic order) is visualized for the same sample. In the case of exchange reactions, negative values correspond to consumption of the metabolite, positive values to production.

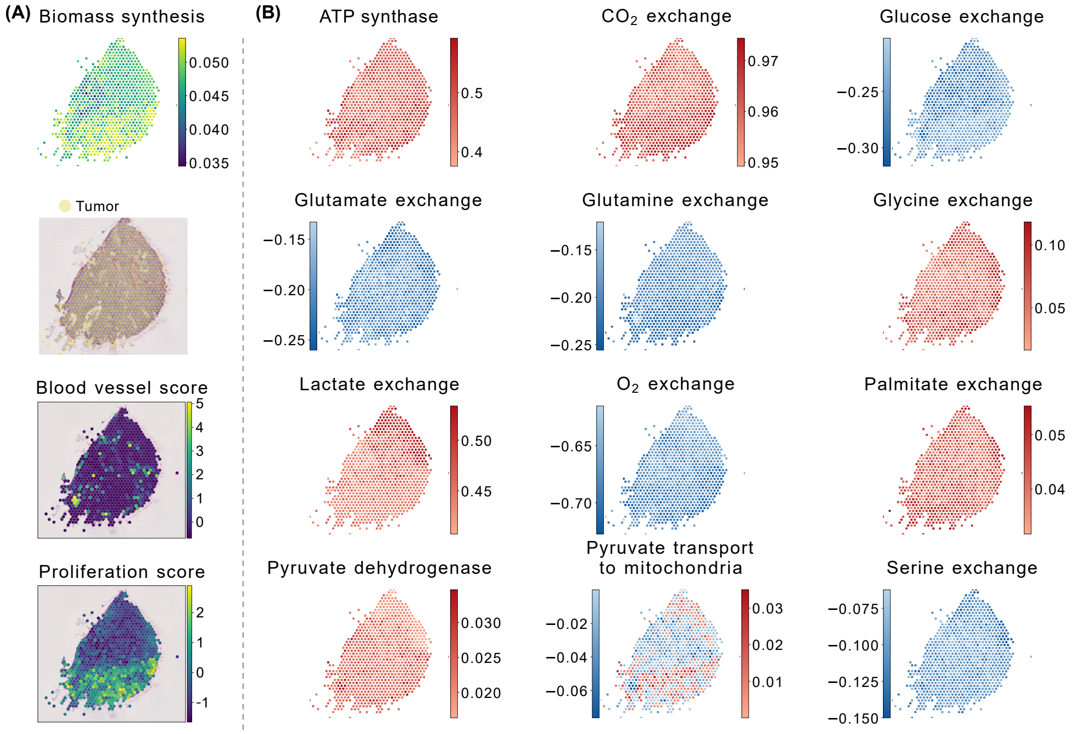

Figure S5: **ccRCC core sample C2.** **(A)** Biomass FES, annotated H&E image, and blood vessel and proliferation scores derived from gene expression. **(B)** The FES of a set of reactions of interest (in alphabetic order) is visualized for the same sample. In the case of exchange reactions, negative values correspond to consumption of the metabolite, positive values to production.

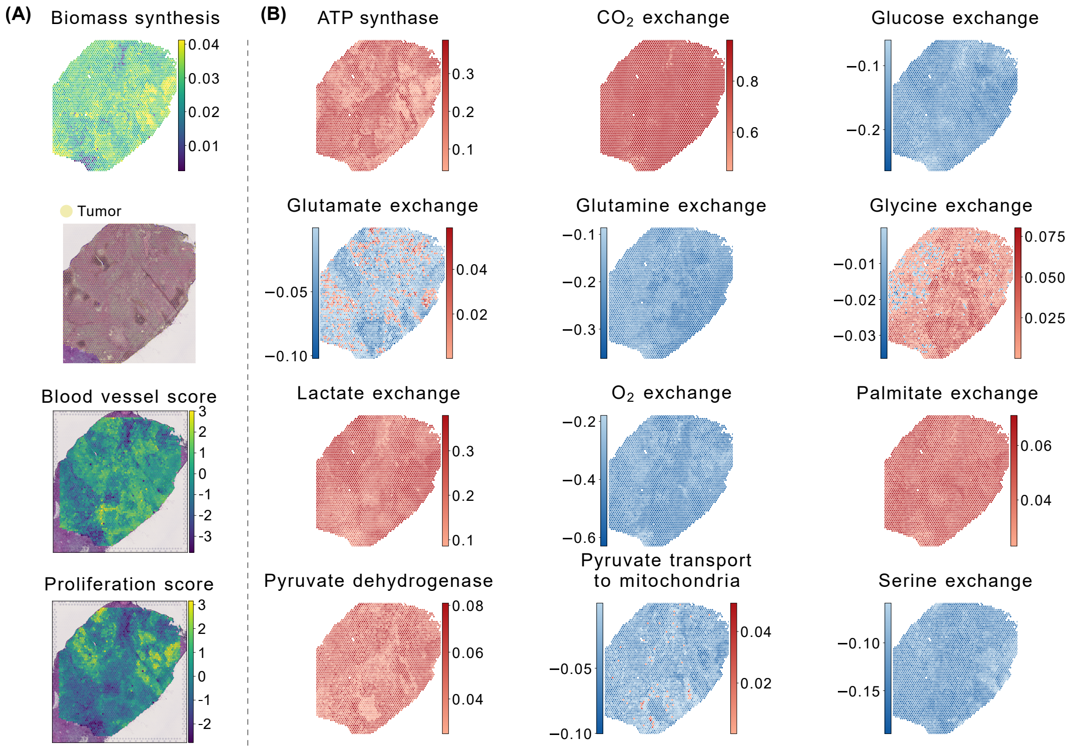

Figure S6: **ccRCC core sample C3.** **(A)** Biomass FES, annotated H&E image, and blood vessel and proliferation scores derived from gene expression. **(B)** The FES of a set of reactions of interest (in alphabetic order) is visualized for the same sample. In the case of exchange reactions, negative values correspond to consumption of the metabolite, positive values to production.

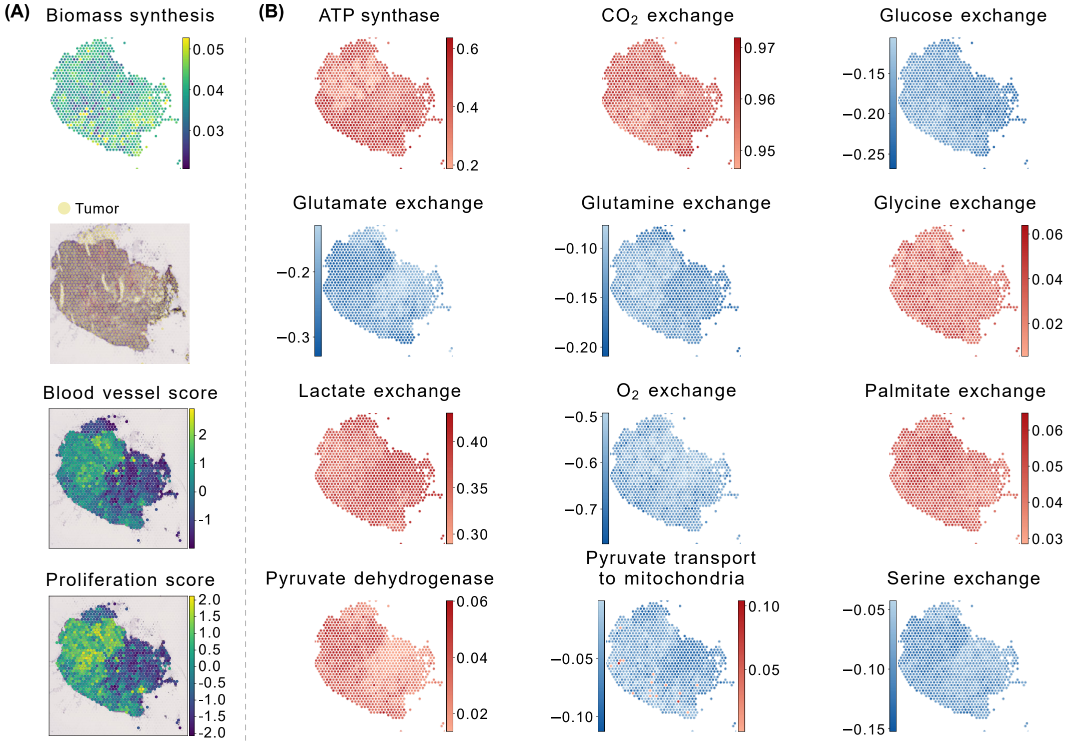

Figure S7: **ccRCC core sample C4. (A)** Biomass FES, annotated H&E image, and blood vessel and proliferation scores derived from gene expression. **(B)** The FES of a set of reactions of interest (in alphabetic order) is visualized for the same sample. In the case of exchange reactions, negative values correspond to consumption of the metabolite, positive values to production.

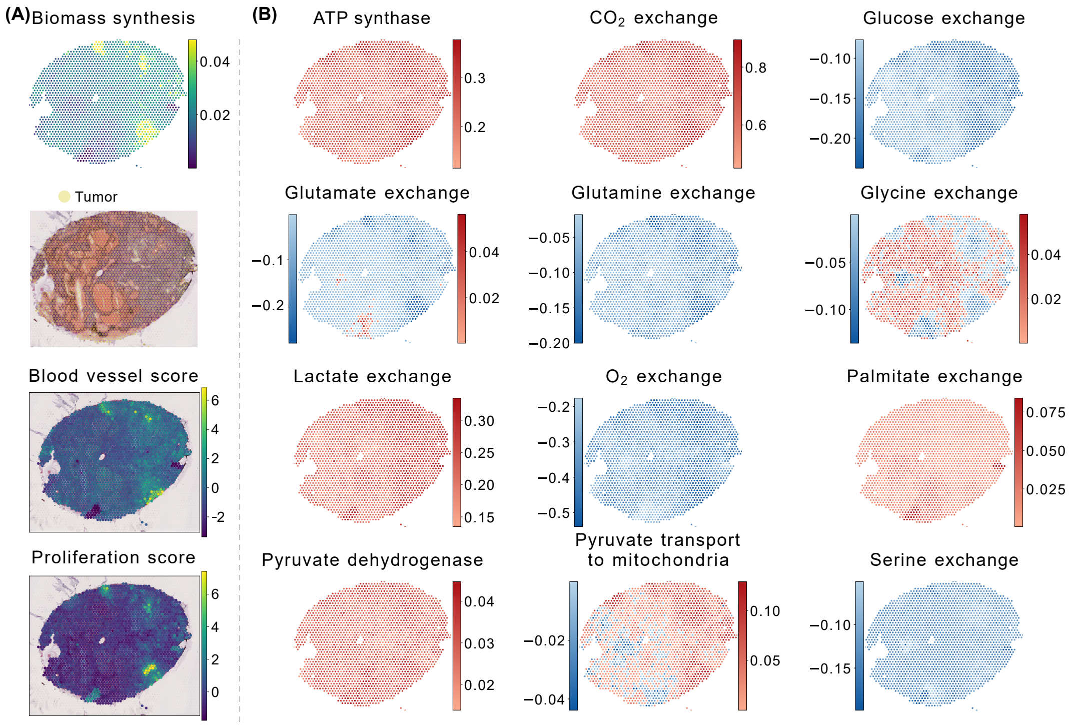

Figure S8: **ccRCC core sample C5.** **(A)** Biomass FES, annotated H&E image, and blood vessel and proliferation scores derived from gene expression. **(B)** The FES of a set of reactions of interest (in alphabetic order) is visualized for the same sample. In the case of exchange reactions, negative values correspond to consumption of the metabolite, positive values to production.

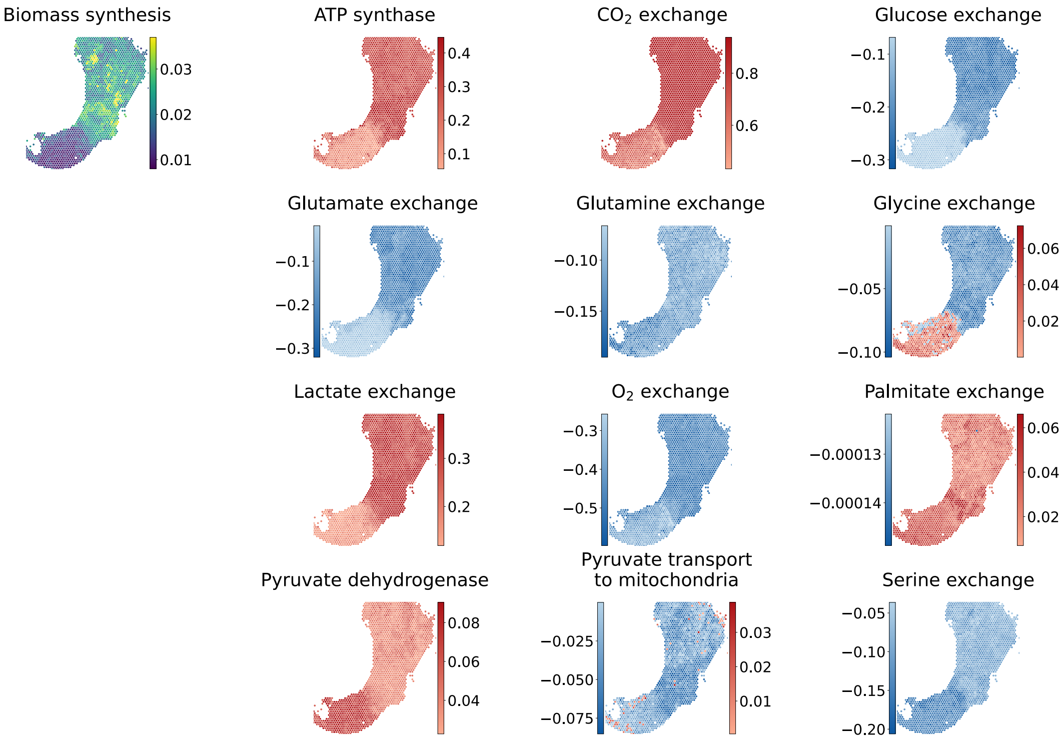

Figure S9: Lower bound of the 99% confidence interval for the FES of a set of reactions of interest ~~(in alphabetic order)~~ is visualized for ccRCC sample I2. In the case of exchange reactions, negative values correspond to consumption of the metabolite, positive values to production.

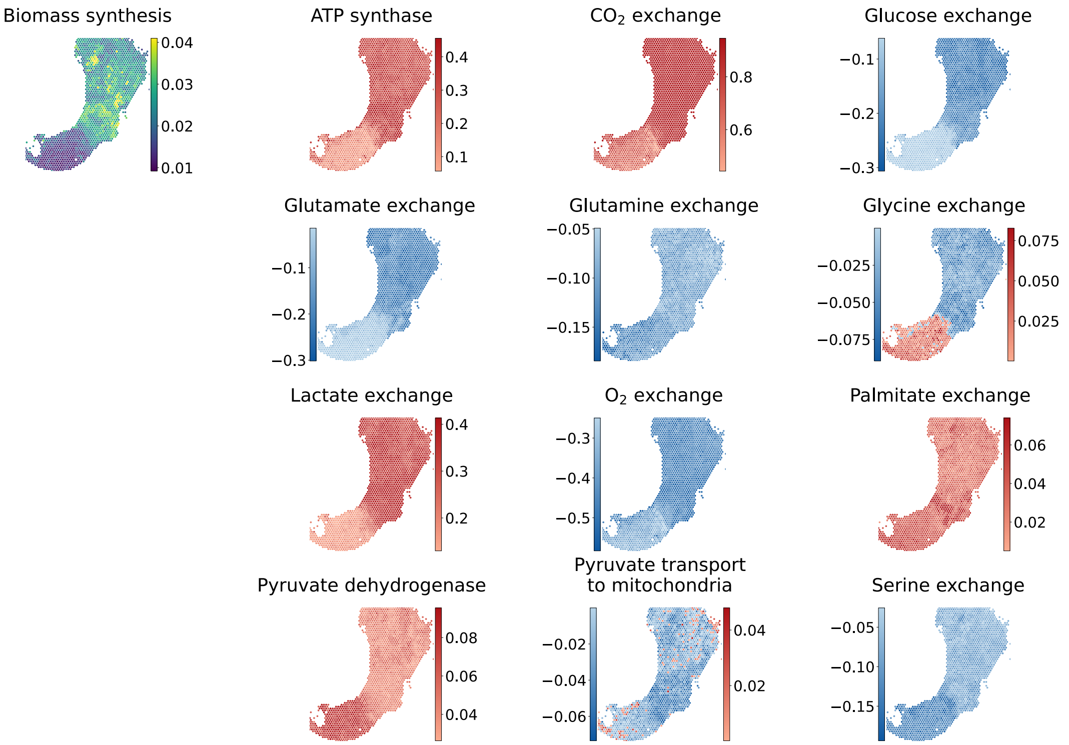

Figure S10: Upper bound of the 99% confidence interval for the FES of a set of reactions of interest ~~(in alphabetic order)~~ is visualized for ccRCC sample I2. In the case of exchange reactions, negative values correspond to consumption of the metabolite, positive values to production.

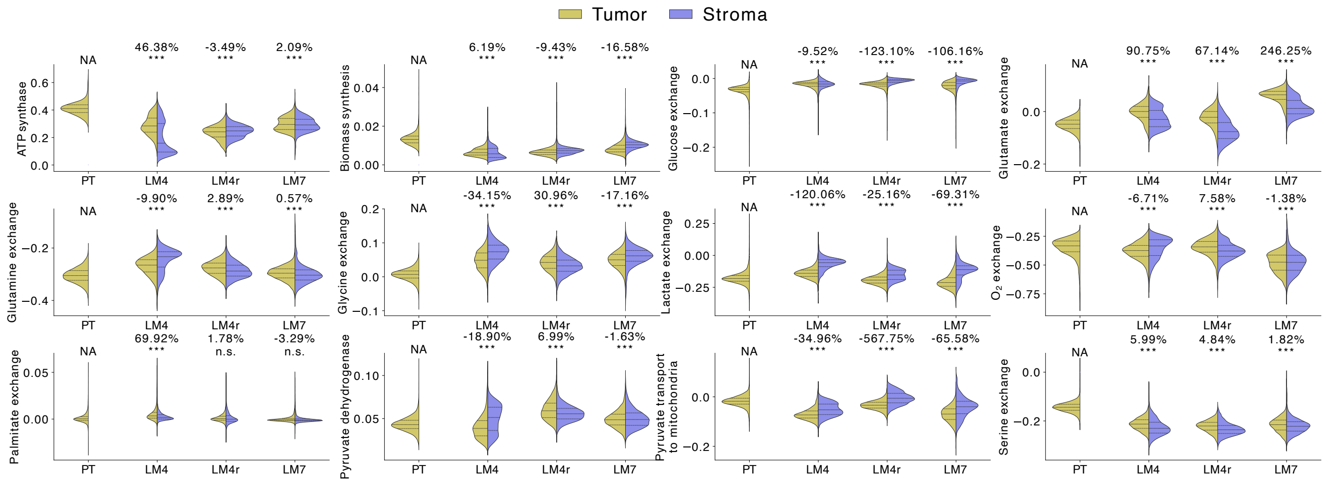

Figure S11: Statistical comparison of the FESs between spots annotated as stroma and those annotated as tumor (based on tissue region annotations) for the selected reactions, listed in alphabetical order. The p-values were calculated using t-tests

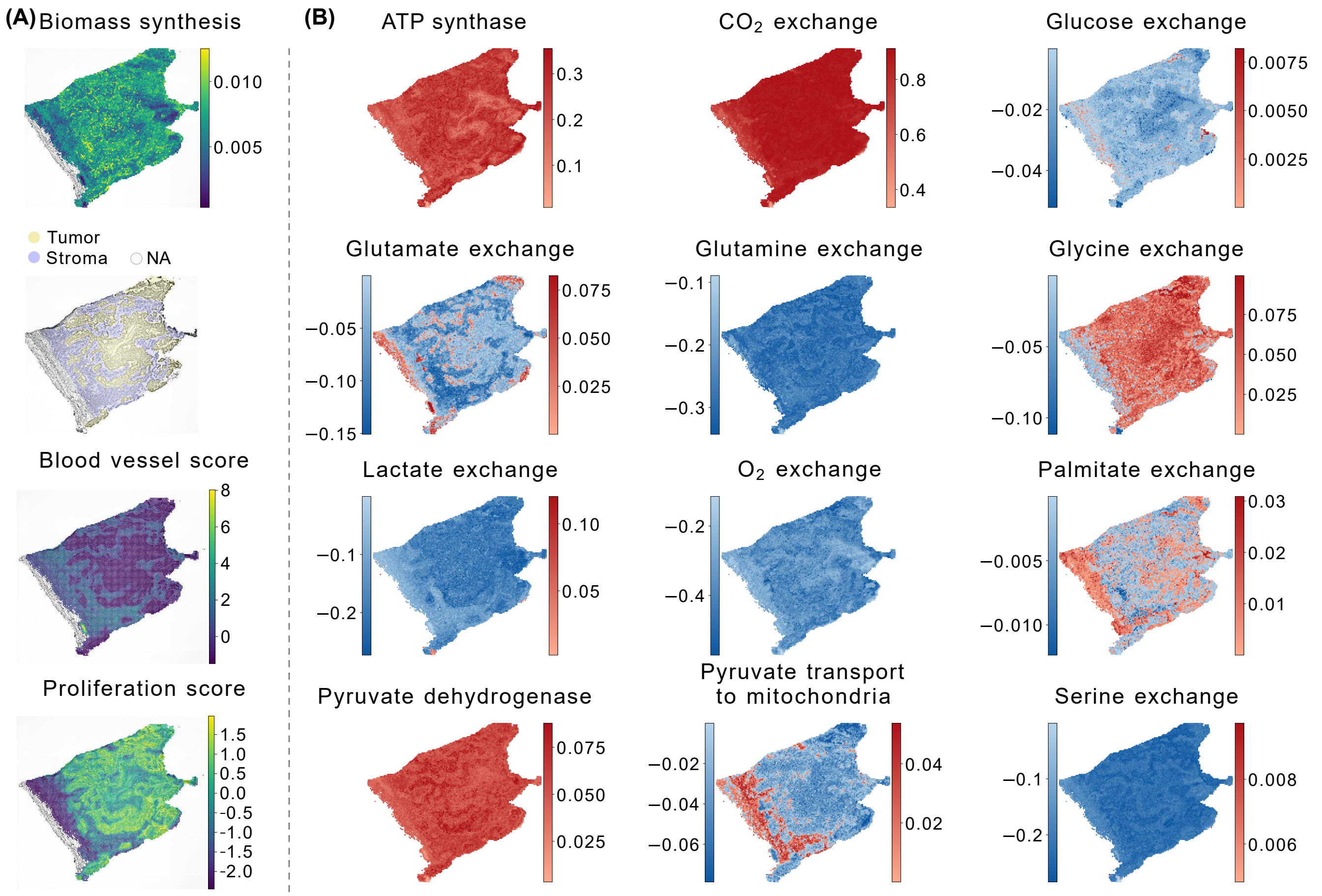

Figure S12: **CRC sample LM4r.** **(A)** Biomass FES, annotated H&E image, and blood vessel and proliferation scores derived from gene expression. **(B)** The FES of a set of reactions of interest (in alphabetic order) is visualized for the same sample. In the case of exchange reactions, negative values correspond to consumption of the metabolite, positive values to production.

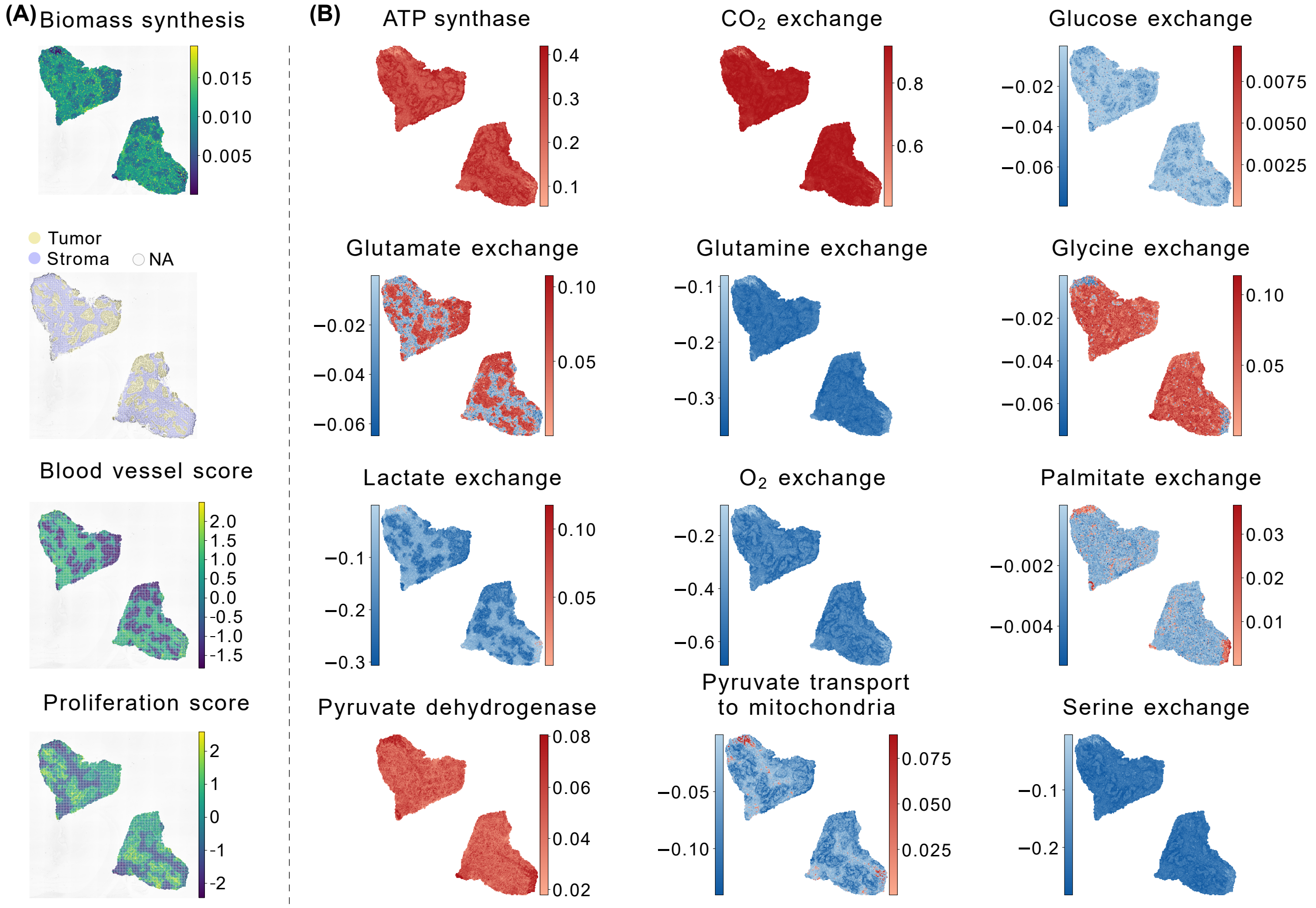

Figure S13: **CRC sample LM7.** **(A)** Biomass FES, annotated H&E image, and blood vessel and proliferation scores derived from gene expression. **(B)** The FES of a set of reactions of interest (in alphabetic order) is visualized for the same sample. In the case of exchange reactions, negative values correspond to consumption of the metabolite, positive values to production.

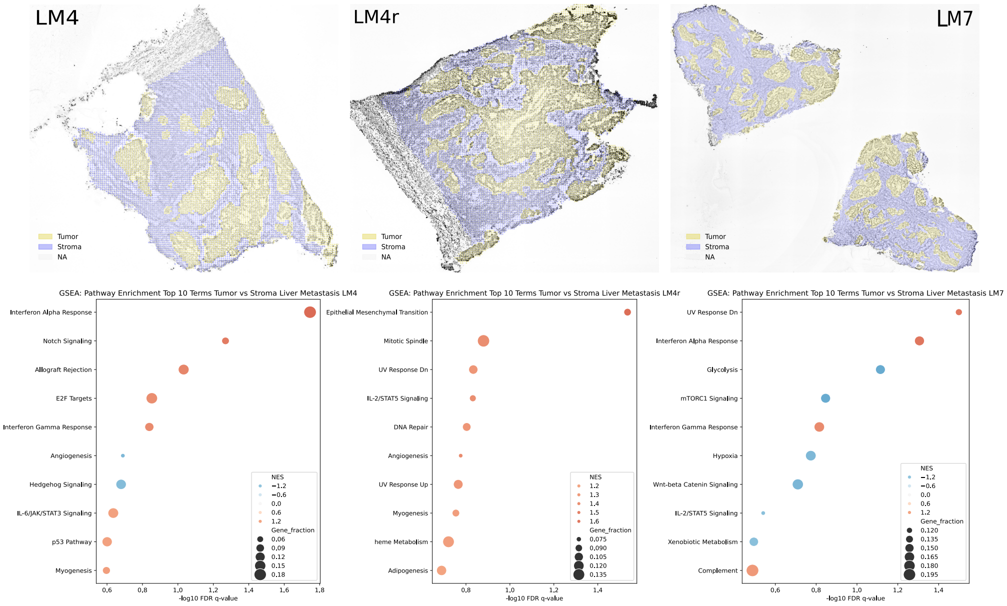

Figure S14: **Pathway enrichment analysis of liver metastasis samples.** Shown are the histological annotations and corresponding dot plots for the gene set enrichment analysis (GSEA) performed using the MSigDB Hallmark gene sets to compare tumor and stroma regions in liver metastasis samples. Positive NES values indicate pathways enriched in tumor regions, whereas negative NES values indicate pathways enriched in stroma. Dot size represents the fraction of detected genes belonging to each pathway. Terms are ordered by FDR q-value.

(A)

(B)

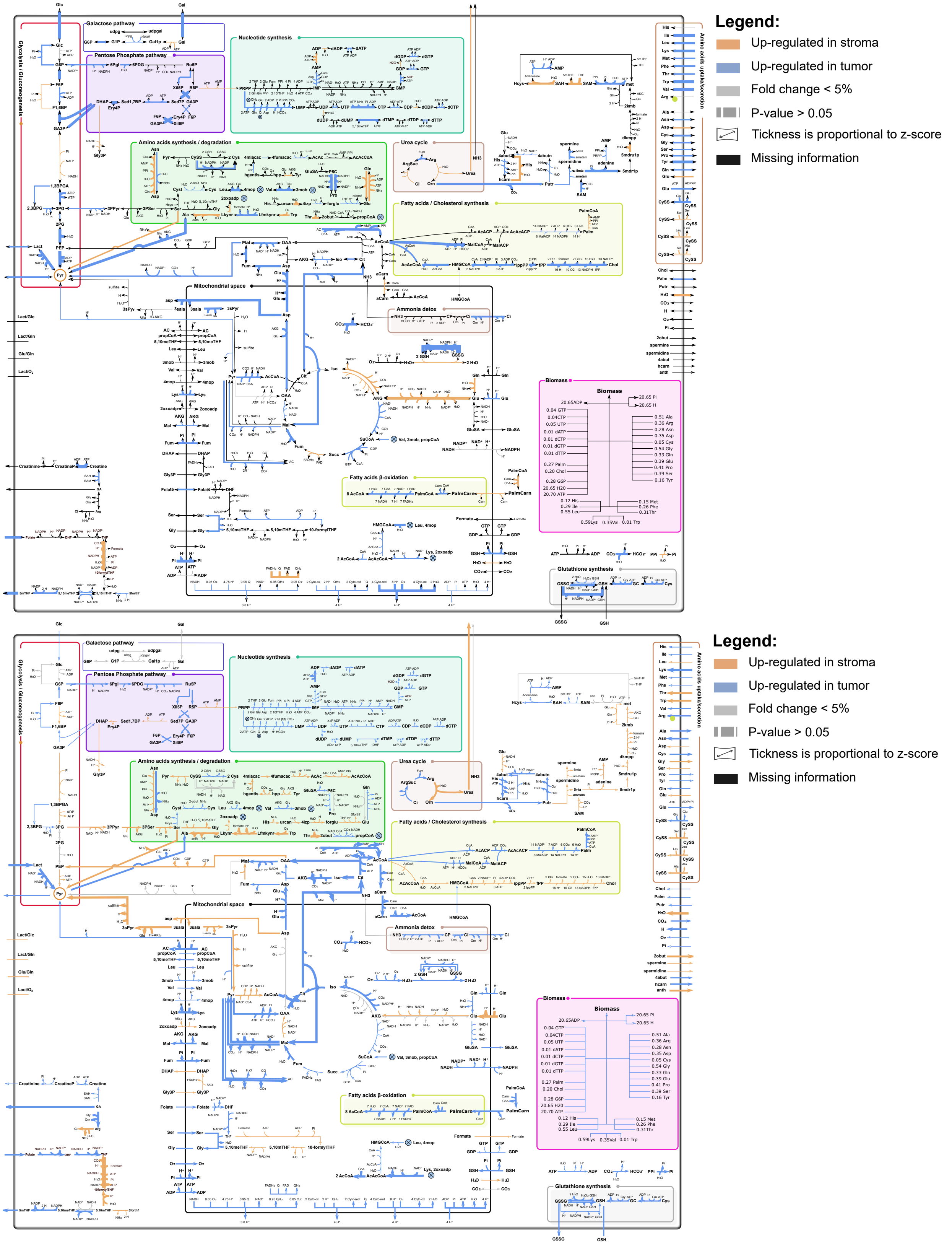

Figure S15: **Stroma vs Tumor.** **(A)** Differences in RAS between stroma and tumor spots in the CRC liver metastasis sample LM4. **(B)** Differences in FESs between stroma and tumor spots in the same sample.

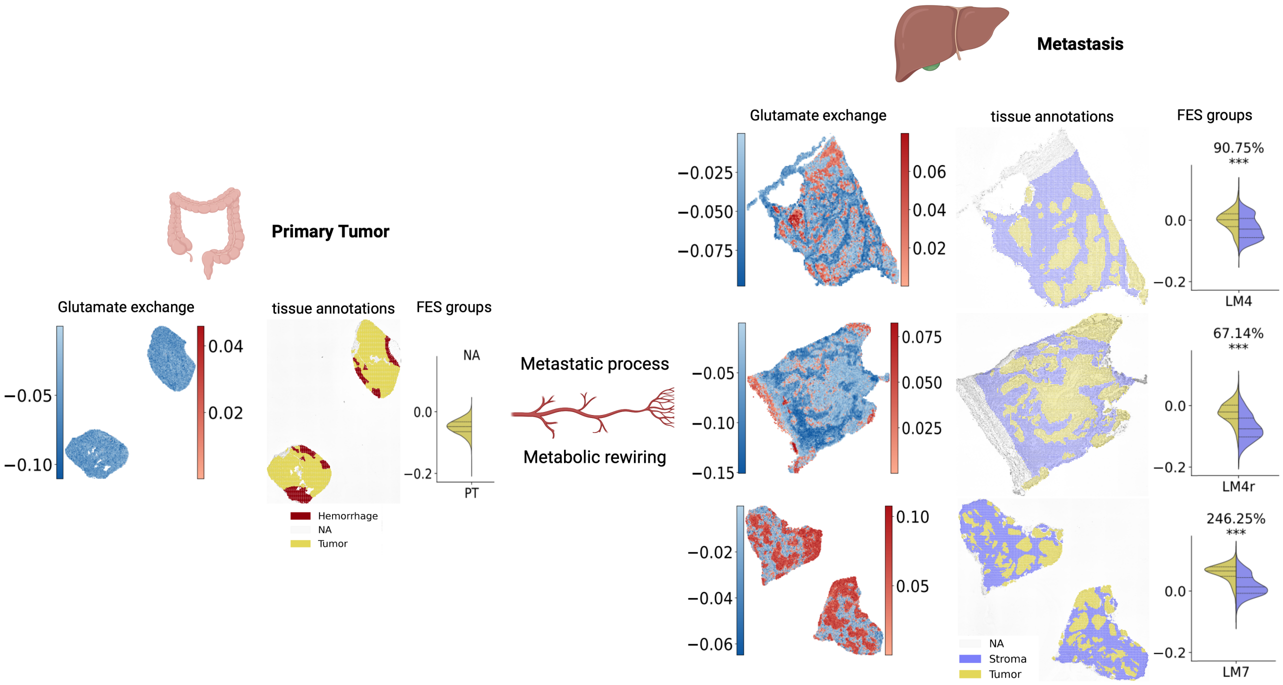

Figure S16: On the left, the violin plot represents glutamate exchange in the primary tumor (PT) region, where stromal tissue is absent, showing uniform glutamate consumption across the tumor without statistical testing. On the right, three metastatic liver samples (LM7, LM4, LM4r) are shown. For each metastasis, spatial maps of glutamate exchange (center) reveal heterogeneity within the tumor region. Black arrows indicate areas at the tumor-stroma interface where glutamate production is detected, in contrast to core regions that predominantly consume glutamate, albeit at a lower rate than stromal cells. Adjacent to the spatial maps, hematoxylin and eosin (H&E)-based tissue annotations highlight tumor (yellow) and stroma (blue). The violin plots on the far right compare stromal and tumor regions for each metastatic sample, with statistical tests indicating significant differences in glutamate exchange between the two compartments

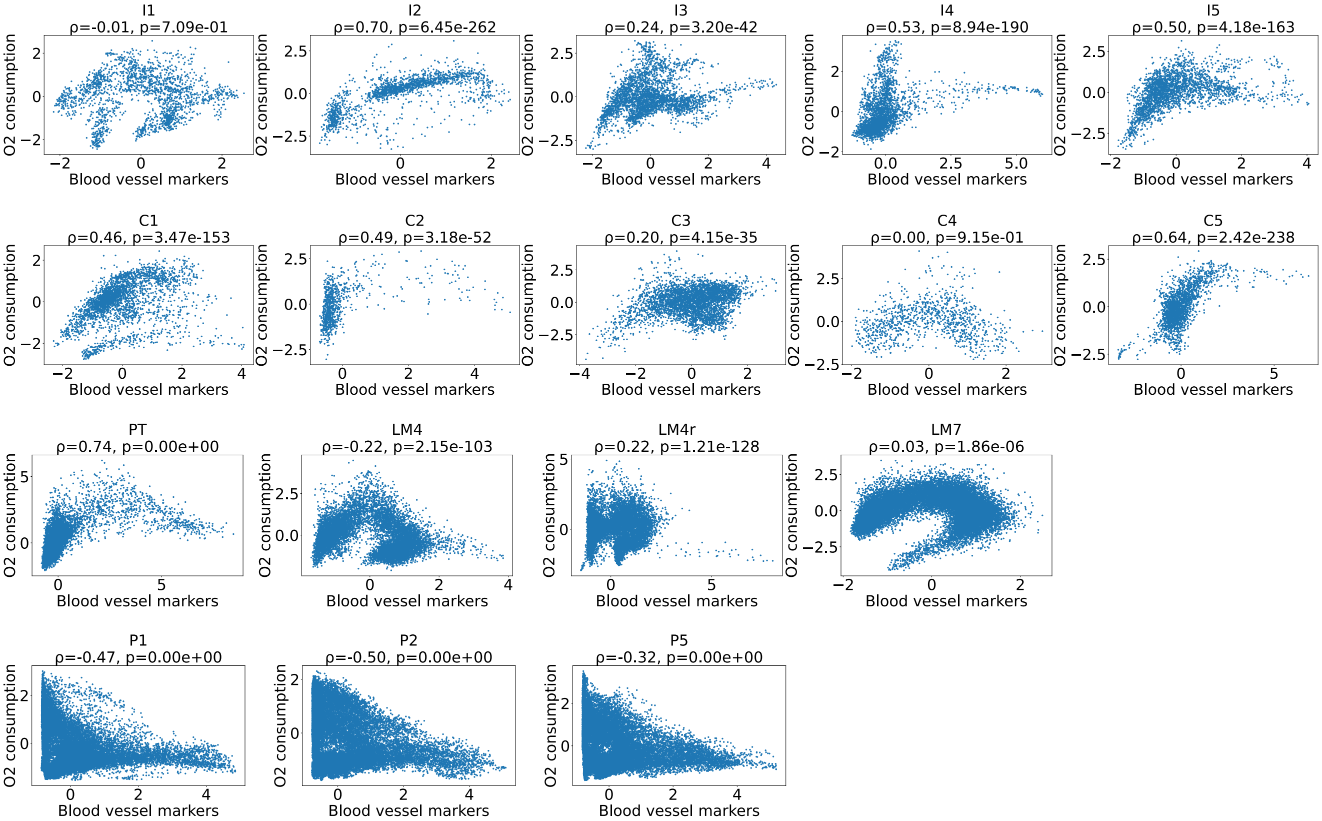

Figure S17: **Vascularization and oxygen consumption.** Scatterplots illustrating the relationship between blood-vessel markers and oxygen consumption across all samples. Each subplot is titled with the corresponding sample name. Spearman correlation was used to compute correlation coefficients and associated p-values.

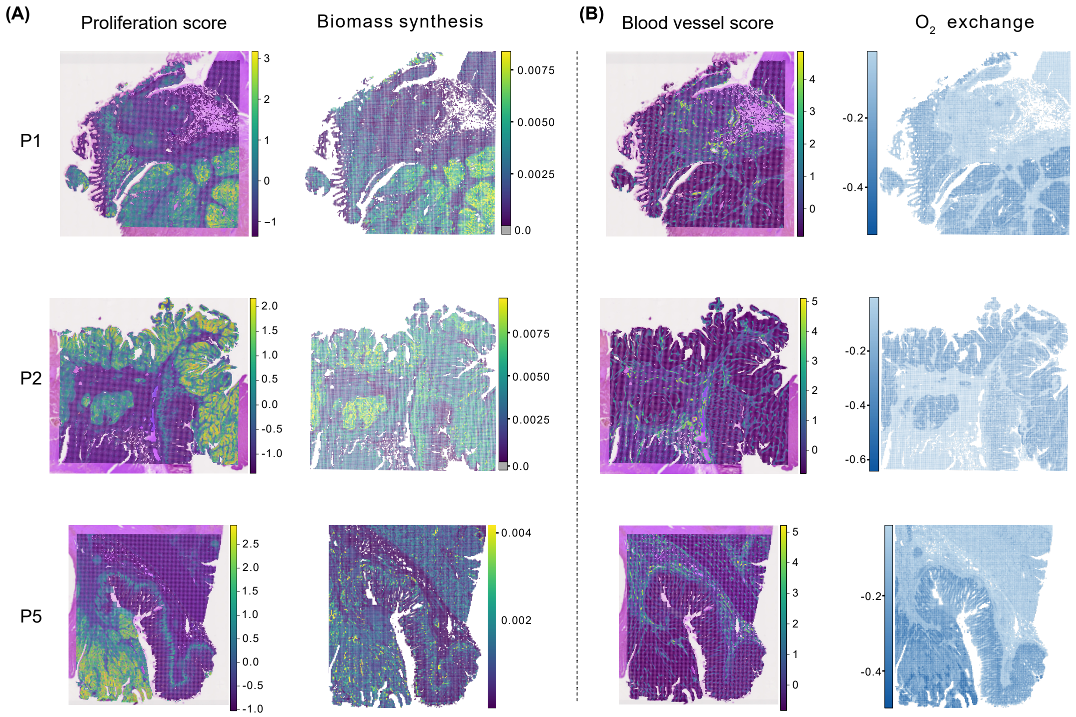

Figure S18: **Spatial distribution of fluxes and biomarkers.** Panel (A-B), rows correspond to sample P1, P2, and P5 from top to bottom. Panel (A), left column shows spatial distribution of proliferation marker score. Right columns shows the spatial distribution of the flux of the biomass production. Panel (B), left column shows spatial distribution of blood vessel marker score. Right columns shows the spatial distribution of the flux of Oxygen consumption.
